# MCR-1-dependent lipid remodelling compromises the viability of Gram-negative bacteria

**DOI:** 10.1080/22221751.2022.2065934

**Published:** 2022-04-28

**Authors:** Siyuan Feng, Wanfei Liang, Jiachen Li, Yong Chen, Dianrong Zhou, Lujie Liang, Daixi Lin, Yaxin Li, Hui Zhao, Huihui Du, Min Dai, Li-Na Qin, Fan Bai, Yohei Doi, Lan-Lan Zhong, Guo-bao Tian

**Affiliations:** a Program in Pathobiology, The Fifth Affiliated Hospital, Zhongshan School of Medicine, Sun Yat-Sen University, Guangdong, People’s Republic of China; b Key Laboratory of Tropical Diseases Control (Sun Yat-Sen University), Ministry of Education, Guangzhou, People’s Republic of China; c School of Laboratory Medicine, Chengdu Medical College, Chengdu, People’s Republic of China; d Guangdong Provincial Key Laboratory of Biotechnology for Plant Development, School of Life Sciences, South China Normal University, Guangzhou, People’s Republic of China; e College of Biology and Food Engineering, Chongqing Three Gorges University, Chongqing, People’s Republic of China; f Department of Histology and Embryology, Zhongshan School of Medicine, Sun Yat-sen University, Guangzhou, People’s Republic of China; g Biomedical Pioneering Innovation Center (BIOPIC), School of Life Sciences, Peking University, Beijing, People's Republic of China; h Beijing Advanced Innovation Center for Genomics (ICG), Peking University, Beijing, People’s Republic of China; i University of Pittsburgh School of Medicine, Pittsburgh, PA, USA; j Department of Microbiology and Infectious Diseases, School of Medicine, Fujita Health University, Aichi, Japan; k School of Medicine, Xizang Minzu University, Xianyang, People’s Republic of China

**Keywords:** MCR-1, outer membrane (OM) permeability, viability, lipid homeostasis, colistin

## Abstract

The global dissemination of the mobilized colistin resistance gene, *mcr-1*, threatens human health. Recent studies by our group and others have shown that the withdrawal of colistin as a feed additive dramatically reduced the prevalence of *mcr-1*. Although it is accepted that the rapid reduction in *mcr-1* prevalence may have resulted, to some extent, from the toxic effects of MCR-1, the detailed mechanism remains unclear. Here, we found that MCR-1 damaged the outer membrane (OM) permeability in *Escherichia coli* and *Klebsiella pneumonia* and that this event was associated with MCR-1-mediated cell shrinkage and death during the stationary phase. Notably, the capacity of MCR-1-expressing cells for recovery from the stationary phase under improved conditions was reduced in a time-dependent manner. We also showed that mutations in the potential lipid-A-binding pocket of MCR-1, but not in the catalytic domain, restored OM permeability and cell viability. During the stationary phase, PbgA, a sensor of periplasmic lipid-A and LpxC production that performed the first step in lipid-A synthesis, was reduced after MCR-1 expression, suggesting that MCR-1 disrupted lipid homeostasis. Consistent with this, the overexpression of LpxC completely reversed the MCR-1-induced OM permeability defect. We propose that MCR-1 causes lipid remodelling that results in an OM permeability defect, thus compromising the viability of Gram-negative bacteria. These findings extended our understanding of the effect of MCR-1 on bacterial physiology and provided a potential strategy for eliminating drug-resistant bacteria.

## Introduction

The emergence of antibiotic-resistant bacteria is a major global public health crisis. Colistin is a last line of defense in the antibiotic treatment of bacterial infections, especially those caused by multiple-drug-resistant bacteria. However, the efficiency of colistin has been limited by the emergence of the mobilized colistin resistance gene, *mcr-1*, in China in 2016 [1]. This gene has been found on numerous plasmids (e.g. IncI2, IncX4, and IncHI2) from diverse pathogen species, and has spread across more than 70 countries on five continents [2–4]. Because colistin is used as a feed additive for animals in many countries [1], it is generally acknowledged that there is a close relationship between the global spread of *mcr-1* and colistin use in animal husbandry. To stop the dissemination of *mcr-1*, China banned colistin sulphate premix as a livestock growth promoter on April 30, 2017. Recent studies by our group and others have shown that the withdrawal of colistin as a feed additive dramatically reduced the prevalence of *mcr-1* [5–9]. Although a better understanding of this phenomenon could provide a potential strategy for eliminating drug-resistant bacteria, the detailed mechanism remains unclear.

MCR-1 is a member of the phosphatidylethanolamine (pEtN) transferase family and transfers a pEtN residue to the lipid-A present in the cell membranes of Gram-negative bacteria [10]. This modified lipid-A shows a low affinity for colistin and polymyxin, reducing the activities of these antimicrobial drugs [10]. Several research groups have presented evidence that MCR-1 profoundly affects the fitness and morphology of bacteria [11–15]. For example, the overexpression of MCR-1 reduces the growth rate, viability, and competitiveness of bacteria and disrupted their cell membranes [16]. Research has also attributed this fitness loss and bacterial membrane to the embedding of the MCR-1 in the *Escherichia coli* outer membrane (OM) and the modification of lipopolysaccharide (LPS) with pEtN [16]. These findings led us to hypothesize that the reduced prevalence of *mcr-1* after the ban on colistin-positive additives resulted from the toxicity of MCR-1. On the contrary, Choi et al. reported that plasmids bearing *mcr-1* did not compromise the fitness of *E. coli* [15]. Moreover, the acquisition of an IncI2-type plasmid increased the fitness of host *E. coli* DH5-α cells [17]. Therefore, there is an ongoing debate about the effects of MCR-1 on bacterial fitness. Notably, natural conditions are rarely ideal for microbial growth, especially compared with the rich media used in laboratory settings. However, the roles of MCR-1 in the growth and fitness of bacteria have been studied under laboratory conditions [11–17], the survival of *mcr-1*-positive bacteria under natural conditions has rarely been considered.

In natural environments, bacteria are often subjected to diverse stresses, including competition with other microbial cohabitants, pH changes, and nutrient depletion. For example, bacteria experience a dramatic change in nutrient abundance as they travel through the host gut, into a less rewarding environment. During nutrient depletion, bacteria enter the relatively quiescent state known as the “stationary phase” [18]. In fact, most microorganisms in the environment are predominantly in the stationary phase [19,20]. Bacteria display morphological changes in response to changes in their nutrient status or the presence of environmental challenges [21]. These stress conditions may cause bacterial shrinkage and macromolecular cellular damage, resulting in cell death [22]. For example, the reduced prevalence of *mcr-1* in humans, animals, and the environment possibly indicates an unfavourable effect of environmental stress on bacterial viability.

Here, we showed that MCR-1 increased the permeability of OM in Gram-negative bacteria and that this event was associated with bacterial cell shrinkage and death in the stationary phase. We also showed that the potential lipid-A-binding pocket of MCR-1, but not its catalytic domain, was responsible for the defect in OM permeability. We also provided evidence that lipid remodelling caused by MCR-1 contributed to the OM permeability defect. Our data provided new insights into the mechanisms underlying the reduced prevalence of *mcr-1*, and suggested new approaches to the control of drug-resistant bacteria.

## Materials and methods

### Bacterial strains and plasmids

The bacterial strains and plasmids used in this study are listed in Supplementary Table S1. The primers used in this study are listed in Supplementary Table S2. *Escherichia coli* carrying plasmid pREDCas9 [23] was cultured at 30°C. Unless otherwise stated, all *E*. *coli* strains were grown in Luria–Bertani (LB) broth or on LB agar plates at 37°C. When indicated, *E. coli* strains were grown in M9 minimal media (M9 + 1% glucose). Antibiotics and other chemicals were used at the following final concentrations: chloramphenicol, 30 µg/mL; colistin, 2 µg/mL; ampicillin, 100 µg/mL; spectinomycin, 100 µg/mL; and bleomycin, 50 µg/mL. Isopropyl-β-d-thiogalactopyranoside (IPTG) was used at a concentration of 0.5 mM.

### Strain construction

A plasmid-harbored *mcr-1* gene was deleted by CRISPR–Cas9-mediated genome editing in *E. coli* DH5α. Briefly, three 20-bp spacer fragments targeting *mcr-1* were digested with *Bsm*BI (Thermo Scientific) and inserted into pgRNA (addgene #44251). To construct the donor DNA, two 500-bp homologous arms and the sfGFP-bleomycin^R^ fusion gene were amplified separately and fused together with fusion PCR. Electrocompetent cells were generated as previously described [23]. Briefly, bacterial cells were resuspended in 1 mL of SOC medium, and after electroporation, were incubated for 3 h at 30°C and then plated on LB agar plates containing 100 µg/mL spectinomycin, 100 µg/mL ampicillin, and 50 µg/mL bleomycin. The mutant strains were confirmed with Sanger (DNA) sequencing. To further confirm the deletion of *mcr-1*, *in vitro* broth microdilution methods were used to evaluate colistin resistance for *mcr-1* mutant strain. As expected, all *mcr-1* mutant strains were susceptible to colistin (Supplementary Table S3).

To construct the IbpA-tetracysteine (TC)-expressing strain, a 20-bp single guide RNA (sgRNA) fragment targeting the *ibpA* stop codon was digested with *Bsm*BI and inserted into *Bsm*BI-digested pgRNA-bacteria. The donor DNA was cloned from the *E. coli* strain BW25113 genomic template with primers IbpA-up-5F/IbpA-TC-UP-3R and IbpA-TC-DO-5F/IbpA-do-3R. Strain BW25113 containing plasmid pREDCas9 was transformed with the donor DNA and pgRNA-ibpA. After electroporation, the bacterial cells were resuspended in 1 mL of SOC and cultured for 3 h at 30°C. The bacteria were plated on LB agar containing 100 µg/mL spectinomycin, 100 µg/mL ampicillin, and 50 µg/mL bleomycin. The mutant strains were confirmed with Sanger (DNA) sequencing.

To construct strain PbgA^HA^, a 20-bp sgRNA fragment targeting the *pbgA* start codon was digested with *Bsm*BI and inserted into pgRNA-bacteria. The donor DNA was cloned from the BW25113 genomic template with primers pbgA-UP-5F/pbgA-UP-3R and pbgA-DO-5F/pbgA-DO-3R. Strain BW25113 containing plasmid pREDCas9 was then transformed with the donor DNA and pgRNA-pbgA. After electroporation, the bacterial cells were resuspended in 1 mL of SOC and incubated for 3 h at 30°C. The bacteria were plated on LB agar plates containing 100 µg/mL spectinomycin, 100 µg/mL ampicillin, and 50 µg/mL bleomycin. The mutant strains were confirmed with Sanger (DNA) sequencing. To cure the cells of plasmids, LB broth containing 0.2% l-arabinose was inoculated with the appropriate colonies, and the bacteria were cultured for 6–8 h or overnight.

To generate pACYC-NP-mcr-1, the *mcr-1* gene and its native promoter were amplified from an *mcr-1*-carrying IncX4 [24] plasmid and cloned into pACYDuet-1, a low-copy number (10–12 copies) plasmid (containing the p15A origin of replication).

A two-step PCR procedure was used to generate pACYC-MCR-1-sfGFP. The open reading frame (ORF) of *mcr-1* with its native promoter was amplified from an *mcr-1*-carrying IncX4 plasmid; *sfgfp* was amplified from pEvolvR-enCas9-PolI3M-TBD (addgene #113077); *mcr-1*–*sfgfp* was generated with overlapping PCR and the fused fragment was cloned into pACYCDuet-1.

To generate pBAD24-lpxC, the *lpxC* gene was amplified from the BW25113 genome a and cloned into pBAD24. All the plasmid constructs were confirmed with Sanger sequencing.

### Site-directed mutagenesis

We constructed all mutants with site-directed mutagenesis, in which a two-step PCR procedure was used to replace the indicated site. Specifically, two simultaneous amplification reactions were performed, and both PCR products were purified with a gel extraction kit (Omega, USA). To obtain full-length mutant fragments, the purified DNA from the first-round PCR was mixed in equimolar concentrations and used as the template for the second-round PCR. After purification, the full-length DNA fragment was digested with FastDigest *Xba*I and FastDigest *Xho*I (Thermo scientific), and the digested fragment was cloned into pACYCDuet-1. Competent *E. coli* BW25113 cells were transformed with the ligation product. The recombinant plasmids were purified, and the *mcr-1* variants were sequenced to confirm any mutations.

### Synthesis of cDNA and quantitative real-time PCR (qPCR)

Exponentially growing bacterial cultures (optical density at a wavelength of 600 nm (OD_600_)    0.4–0.6) were pelleted. After the supernatant was removed, the cell pellet was resuspended in RNA-easy Isolation Reagent (Vazyme). The total RNA was precipitated by the addition of isopropanol and collected by centrifugation. The supernatant was discarded, and the RNA pellet was washed with 75% ethanol. After the pellet was air-dried, the mRNA was dissolved in RNase-free H_2_O. Any contaminating genomic DNA was digested with gDNA wiper Mix (Vazyme). The purified mRNA was reverse transcribed to cDNA with HiScript II qRT SuperMix (Vazyme). The cDNA levels of the target genes were then quantified with quantitative real-time PCR (qPCR) on CFX Opus 96 Real-Time PCR Instrument (Bio-Rad) using AceQ Universal SYBR qPCR Master Mix (Vazyme). All qPCR primers were determined to be >95% efficient, and the cDNA molecular masses were experimentally confirmed to be within the linear dynamic range of the assay. The signals were normalized to those of the housekeeping 16S rRNA transcript and quantified with the ΔΔCt method. The error bars are the 95% confidence intervals of three technical replicates.

### Antimicrobial susceptibility testing

The minimum inhibitory concentration (MIC) of antimicrobial agents for *E. coli* BW25113 carrying the *mcr-1* mutant was determined with the broth microdilution method, as previously described [25].

### Checkerboard assay

Checkerboard synergy testing was conducted by broth microdilution. SDS detergent in combination with vancomycin against Vector cells and MCR-1-expressing cells. The fractional inhibitory concentration (FIC) for each drug was calculated by dividing the concentration of drug in the presence of codrug in a combination for a well showing < 10% growth. The interpretation of the FIC was applied as follows: synergy, ≤0.5; indifference, >0.5 to ≤4.0; antagonism, >4.0.

### Sensitivity assays

Bacterial cells were grown overnight in LB broth at 37°C. After the cell density was normalized (OD_600 _= 0.5), the resulting cultures were serially diluted (10^−1^–10^−5^), and 5 μL of each dilution was spotted onto LB agar or LB agar supplemented with the indicated concentrations of sodium dodecyl sulphate (SDS) and ethylenediaminetetraacetic acid (EDTA) (0.5% SDS + 1 mM EDTA or 1% SDS + 1 mM EDTA) [26]. The plates were incubated at 37°C and photographed after ∼24 h.

### Growth kinetics

The *E. coli* strains were grown to mid-log phase (OD_600_ = 0.6–0.8) in LB medium. The precultures were pelleted at 3000 × *g* for 10 min and washed once with phosphate-buffered saline (PBS). The cultures were then adjusted to an OD_600_ of 0.5 with PBS, and diluted at a ratio of 1:10. For each strain, 20 μL diluted culture was added into 180 μL of LB medium in a 96-well plate. When indicated, 20 μL diluted culture was added into M9 minimal media (M9 + 1% glucose). A microplate reader (BioTek) was used to measure the optical density of each well every 30 min. Three replicates were used for each strain. Growth curves were plotted using GraphPad Prism.

### Flow cytometry

Cells were centrifuged at 10,000 × *g* for 5 min and resuspended in 95 μL of 1× Assay Buffer (Sangon). The cells were stained with propidium iodide (PI) for 10 min and sorted with flow cytometry (at least 8000 cells acquired; Beckman Gallios). The data were analysed with the Kaluza software (Beckman Coulter Life Sciences) and visualized with GraphPad Prism.

### Cell staining for fluorescence microscopy

TC-FlAsH™ staining was performed as previously described [27]. Briefly, cells were harvested, washed three times with PBS, and treated with 10 mM EDTA for 15 min to improve membrane permeability. The cells were then resuspended in PBS supplemented with 8 mM FlAsH-EDT2 (Invitrogen) and incubated for 50 min in the dark at 37°C. The FlAsH-EDT2 was washed off.

### Bright-field and fluorescence microscopy

Bright-field and fluorescence imaging were performed on an inverted microscope (Olympus BX63). Illumination was provided by different solid-state lasers at a wavelength of 488 nm for FlAsH. According to the excitation and emission spectra of each fluorophore, the appropriate filter sets were selected. The Fiji/ImageJ software was used to analyse the cell length and fluorescence.

### Super-resolution microscopy

Bacteria were grown in chloramphenicol-containing LB broth. The log-phase bacteria were pelleted at 6000 × *g* for 5 min and washed twice with PBS. The bacterial pellet was then resuspended in 100 μL of PBS, and 2 μL of each suspension was deposited on top of a 1% agarose pad (prepared with M9 medium). After incubation for 2 min to allow the bacteria to settle onto the agarose pad, the pad was covered with a glass coverslip, and the samples were observed immediately with super-resolution microscopy (Nikon, N-SIM, 100×). A 488-nm laser was used to activate the sfGFP protein. All data were analysed with NIS-Elements AR.

### Western blotting analysis

Stationary-phase cells were centrifuged at 7000 × *g* for 10 min and washed twice with PBS. The cells were resuspended in 500 μL of lysis buffer (Beyotime) containing 0.5 μL of Protease Inhibitor Cocktail (Sigma-Aldrich). The cell lysates were then prepared by ultrasonication. Debris was removed with centrifugation. The proteins were separated with SDS-polyacrylamide gel electrophoresis (PAGE) and transferred to a polyvinylidene difluoride (PVDF) membrane. The membrane was incubated with primary antibodies directed against hemagglutinin (anti-HA, clone 3724T; Cell Signaling), LpxC (Cusabio), and RpoB (BioLegend) and secondary antibodies directed against rabbit IgG (Zen Bioscience) and mouse IgG (Dingguo Biology). The proteins were visualized with the ChemiDoc Touch Imaging System (Bio-Rad Laboratories).

### Morphological analysis with transmission and scanning electron microscopy

The samples were prepared as previously described [16]. Log-phase cells were centrifuged at 7000 × *g* for 10 min and washed twice with PBS. The cells were resuspended in electron microscope fixative (Wuhan Sevier Biotechnology Co.). Digital images were captured with a Megaview III digital camera and AnalySIS (Soft Imaging System GmbH, Germany).

The morphological characteristics of the bacteria were detected with scanning electron microscopy, using a field emission scanning electron microscope (S-4800, Hitachi, Tokyo, Japan).

### Structural modelling

The architecture of full-length MCR-1 was modelled with Swiss-Model (https://swissmodel.expasy.org/interactive/LTyGn6/models/). Phosphoethanolamine transferase EptA of *Neisseria meningitidis* (PDB: 5FGN.1.A) was used as the structural template, and the ribbon structure was generated with the PyMol software.

### Statistical analysis

All statistical analyses were performed with GraphPad Prism (version 8.3.0; GraphPad Software). Statistical significance was assessed with Welch’s *t*-test, an unpaired *t*-test, one-way ANOVA followed by Dunnett’s multiple comparison test, or two-way ANOVA followed by Turkey’s multiple comparisons test, as appropriate. The statistical tests used, the exact *p* values, and the sample sizes are shown in each figure legend. A *p* value of ≤ 0.05 was considered statistically significant.

## Results

### MCR-1 damages OM permeability in E. coli and Klebsiella pneumonia

**Figure 1.**
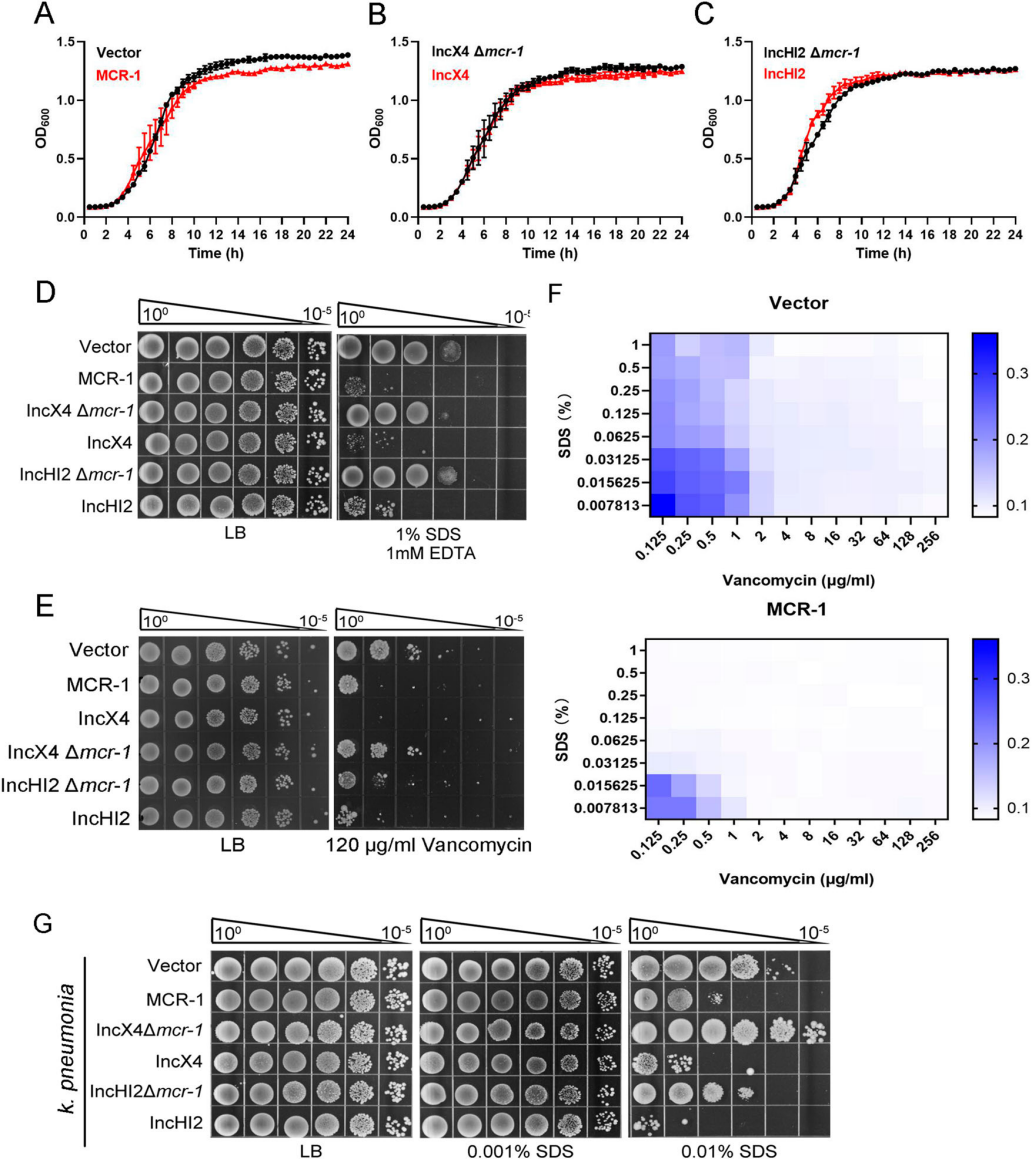
MCR-1 confers an outer membrane permeability defect. (A–C) Growth curves of BW25113 carrying empty vector or expressing MCR-1 (A); BW25113 carrying IncX4 and BW25113 carrying IncX4 Δ*mcr-1* (B); BW25113 carrying IncHI2 and BW25113 carrying IncHI2 Δ*mcr-1* (C). The *y*-axes show optical densities at 600 nm (OD_600_) of broth cultures; *x*-axes show period of growth (hours). (D) Efficiency of plating assays on LB agar plates containing 1% SDS and 1 mM EDTA. Ten-fold dilutions of cultures are indicated above the left plate. (E) Efficiency of plating assays on LB agar plates with 120 μg/mL vancomycin. Ten-fold dilutions of cultures are indicated above the left plate. MCR-1-expressing cells exhibited increased sensitivity to vancomycin. (F) Checkerboard broth microdilution assays showing dose-dependent potentiation of SDS/EDTA by vancomycin against Vector cells (upper) and MCR-1-expressing cells (down). Dark blue regions represent higher cell density. (G) Serial dilutions of the indicated strains were spotted onto LB agar plates or LB agar plates supplemented with SDS. *Klebsiella pneumonia* strains spotted on agar plates were grown at 37°C.

Overexpression of MCR-1 caused has been associated with decreased growth rate, cell viability, competitiveness, and bacterial membrane disruption [16]. Therefore, it has been generally postulated that the reduced prevalence of *mcr-1* may have resulted from the toxicity of MCR-1 [9]. To clarify the impact of MCR-1 on the bacterial physiology, the plasmid pACYC-NP-mcr-1, a low-copy number pACYCDuet-1 derivative carrying *mcr-1* and its native promoter was generated. We then constructed two strains, strain BW25113 carrying pACYC-NP-mcr-1 (MCR-1-expressing cells) and BW25113 carrying the empty vector (Vector cells). Given that IncHI2 and IncX4 are the major plasmid types driving the dissemination of *mcr-1*, two mutant strains lacking *mcr-1*, BW25113 IncX4 Δ*mcr-1* and BW25113 IncHI2 Δ*mcr-1*, were generated with CRISPR–Cas9-mediated genome editing [23] for further confirmation of MCR-1-mediated phenotype. Next, bacterial growth and cellular morphology were examined in the presence or absence of MCR-1 in LB media. We found that MCR-1 expression did not affect bacterial growth (Figure 1(A–C)) and both strains showed distinct, well-structured OMs, and no damaged bacterial membrane was observed in MCR-1 cells (Figure S1). Surprisingly, all the *mcr-1*-positive cells displayed increased sensitivity to detergents compared with the *mcr-1*-negative cells (Figure 1(D) and Figure S2A). Moreover, MCR-1-expressing cells were sensitive to vancomycin and rifampicin, in contrast to *mcr-1*-negative cells (Figure 1(E) and Figure S2B). Furthermore, a lower concentration of SDS is capable of potentiating vancomycin into MCR-1-expressing cells (Figure 1(F)), suggesting that MCR-1 generally increased the permeability of OM in *E. coli*.

We wondered whether this phenotype caused by MCR-1 might exist in *K. pneumonia*, one of the main hosts of *mcr-1*. To test this, we constructed *K. pneumonia* strains, including *K. pneumonia* ATCC 138833 carrying the empty vector, *K. pneumonia* ATCC 138833 carrying pACYC-NP-mcr-1, *K. pneumonia* ATCC 138833 carrying IncX4, *K. pneumonia* ATCC 138833 carrying IncX4 Δ*mcr-1*, *K. pneumonia* ATCC 138833 carrying IncHI2, and *K. pneumonia* ATCC 138833 carrying IncHI2 Δ*mcr-1*, and measured their OM permeability. As expected, all the *mcr-1*-positive strains showed increased sensitivity to SDS (Figure 1(G)), indicating that MCR-1 also rendered the OM defective in *K. pneumonia*.

### MCR-1 induces cell shrinkage and death in the stationary phase and reduces the ability to recover

During the stationary phase, microbial populations undergo shrinkage, damaged macromolecular components, and cell death [22]. Because the prevalence of *mcr-1* in humans, animals, and the environment decreased significantly after the withdrawal of colistin as a feed additive, we initially predicted a link between a stressful environment and the fate of *mcr-1*-positive bacteria.

**Figure 2.**
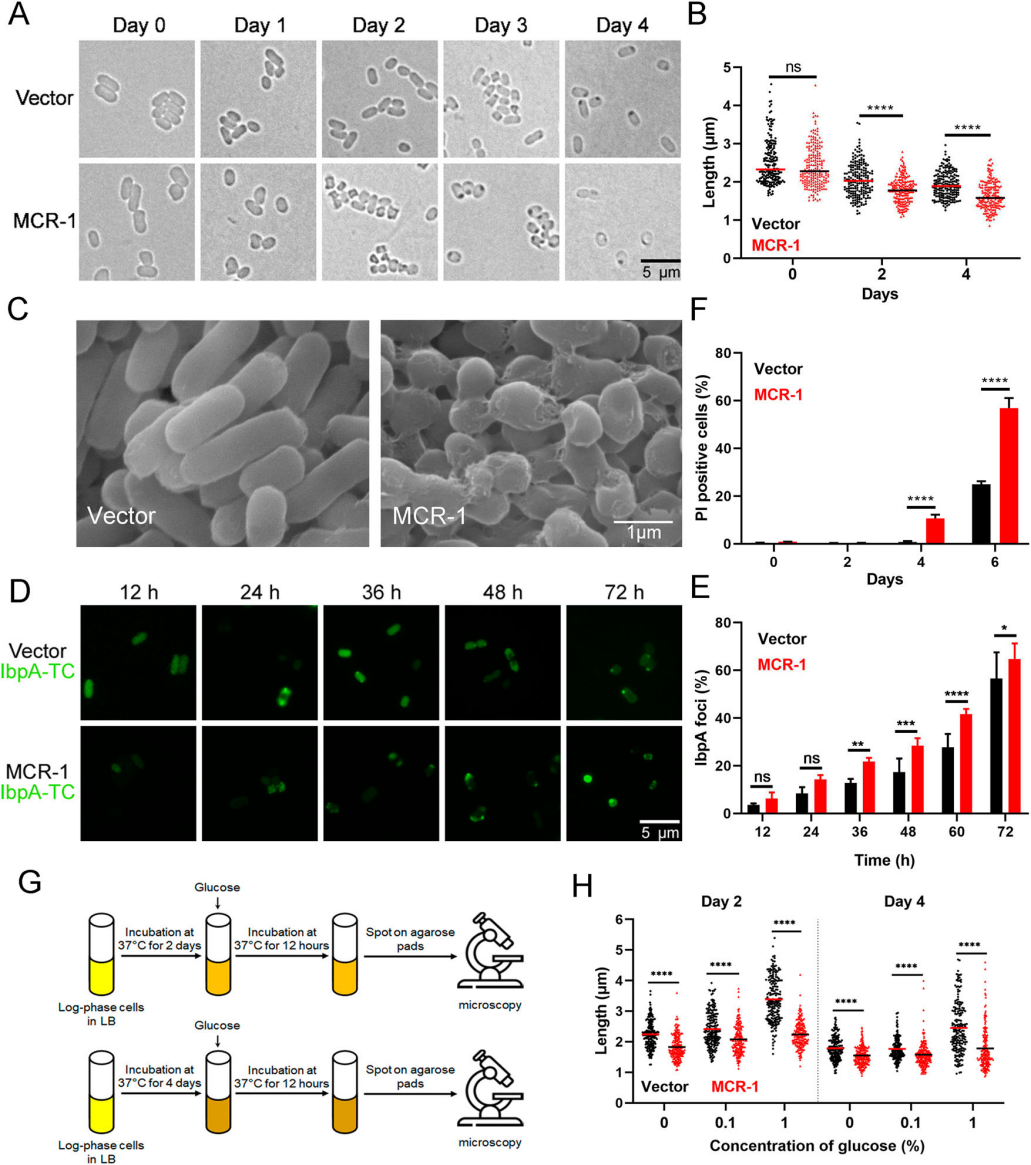
MCR-1 triggers bacterial shrinkage and cell death. (A) Bright-field images of BW25113 carrying empty vector or BW25113 expressing MCR-1 showing morphology variant were induced during the stationary phase (>200 cells for each group). (B) Scatterplots of cell length in Vector cells (black) or MCR-1-expressing cells (red) at the indicated time points. Middle lines represent median values. (C) SEM micrographs of BW25113 carrying empty vector (left) or BW25113 carrying pACYC-NP-mcr-1 (right). Bacterial samples were collected for imaging after 4 days in the stationary phase. (D) Fluorescent images of cells displaying protein aggresomes after they were induced in the stationary phase. Protein aggregates were labelled with IbpA-TC-FlAsH. At the indicated culture times, the cells were stained with FlAsH and washed with PBS before imaging. (E) Percentages of cells with IbpA-TC-FlAsH foci at the indicated times. (F) Percentage of dead cells was evaluated with flow cytometry. The cells were stained with PI after the indicated times in culture and washed with PBS before flow cytometry. (G) Experimental schema for testing the ability of cells to recover. Log-phase cells were cultured in LB broth for 2 days (upper) or 4 days (lower), and the bacterial cultures were then supplemented with increasing concentrations of glucose. The bacteria were photographed after supplementation for 12 h. (H) Scatterplots of cell lengths in Vector cells (black) and MCR-1-expressing cells (red) at the indicated time points. Middle lines represent median values (*n* = 3 biological replicates per group; data are means ± SEM, assessed with two-way ANOVA and Tukey’s multiple comparison test; ***p < 0.001, ****p < 0.0001 compared with the Vector group; ns, no significance).

To test this hypothesis, we examined the role of MCR-1 in bacterial growth in minimal media. It appeared that MCR-1 slightly impaired bacterial growth in M9 minimal media (Figure S3). Next, Vector cells and MCR-1-expressing cells were cultured to stationary phase. We observed that both the strains showed the same length at day 0 (Figure 2(A)). However, the length was significantly shorter in MCR-1-expressing cells than in Vector cells at day 2 and day 4 (Figure 2(B)). Consistent with this observation, further analysis of the integrity of the bacterial membrane with SEM revealed that the MCR-1-expressing cells showed shrinkage and more-severe membrane damage in the stationary phase than the Vector cells (Figure S4A and Figure 2(C)). This indicated that MCR-1 induced bacterial shrinkage during starvation. We also used TEM to examine the cellular morphology of *mcr-1*-positive *K. pneumonia* in stationary phase. As expected, the periplasm was enlarged at one pole of the MCR-1-expressing cells but was not in the Vector cells (Figure S4B). Whereas most hyperosmotic-shocked cells displayed periplasmic shrinkage at both poles, starvation-induced shrinkage occurred predominantly at one pole [28]. Our result indicated that as in *E. coli*, MCR-1 also promoted starvation-induced shrinkage in *K. pneumonia*.

Interestingly, we noted a high percentage of dark foci, indicating protein aggresomes, in the *mcr-1*-positive cells, and fewer in the cells containing the empty vector (Figure 2(A)). To confirm the effect of MCR-1 on protein aggregation, we labelled IbpA, a small chaperone that is closely associated with protein aggregates in *E. coli*, with TC-FlAsH [29]. As expected, the number of IbpA-TC-FlAsH foci was significantly higher in the MCR-1-expressing cells than in the Vector cells at the early stage of the stationary phase (Figure 2(D,E)). Therefore, we postulated that MCR-1 was detrimental to bacterial viability during the stationary phase. Cell viability was then determined with flow cytometry after PI staining. After 2 days, cell viability was high in both strains. However, there were more PI-positive (dead) cells among the MCR-1-expressing cells than among the Vector cells on days 4 and 6 (Figure 2(F)). Together, these results demonstrated that MCR-1 expression caused cell shrinkage and death in stationary phase.

In the stationary phase, OM permeabilization may contribute to bacterial death [30]. Therefore, we hypothesized that the bacterial death caused by MCR-1 results from increased OM permeability. It is well known that extracellular Ca^2+^ stabilizes the OM [30,31]. We supplemented LB with 5 mM Ca^2+^ and measured the cell lengths. Adding Ca^2+^ partly inhibited the shrinkage of the *mcr-1*-positive cells (Figure S5), supporting the notion that the MCR-1-mediated OM permeability defect facilitates bacterial death.

A recent study showed that bacterial shrinkage was a general response to the depletion of useful nutrients [28]. Therefore, we speculated that MCR-1-mediated shrinkage might be rescued by the addition of glucose. To test this, we supplemented LB with increasing concentrations of glucose and measured the cell lengths (Figure 2(G)). On days 2 and 4, the cell length of BW25113 carrying the empty vector increased significantly after supplementation with 1% glucose, whereas the *mcr-1*-positive cells only displayed a slight increase (Figure 2(H)), indicating that the viability of *mcr-1*-positive cells, which showed significant loss of fitness, could not be restored by supplementation with glucose.

### Catalytic activity of MCR-1 negligibly affects bacterial viability

**Figure 3.**
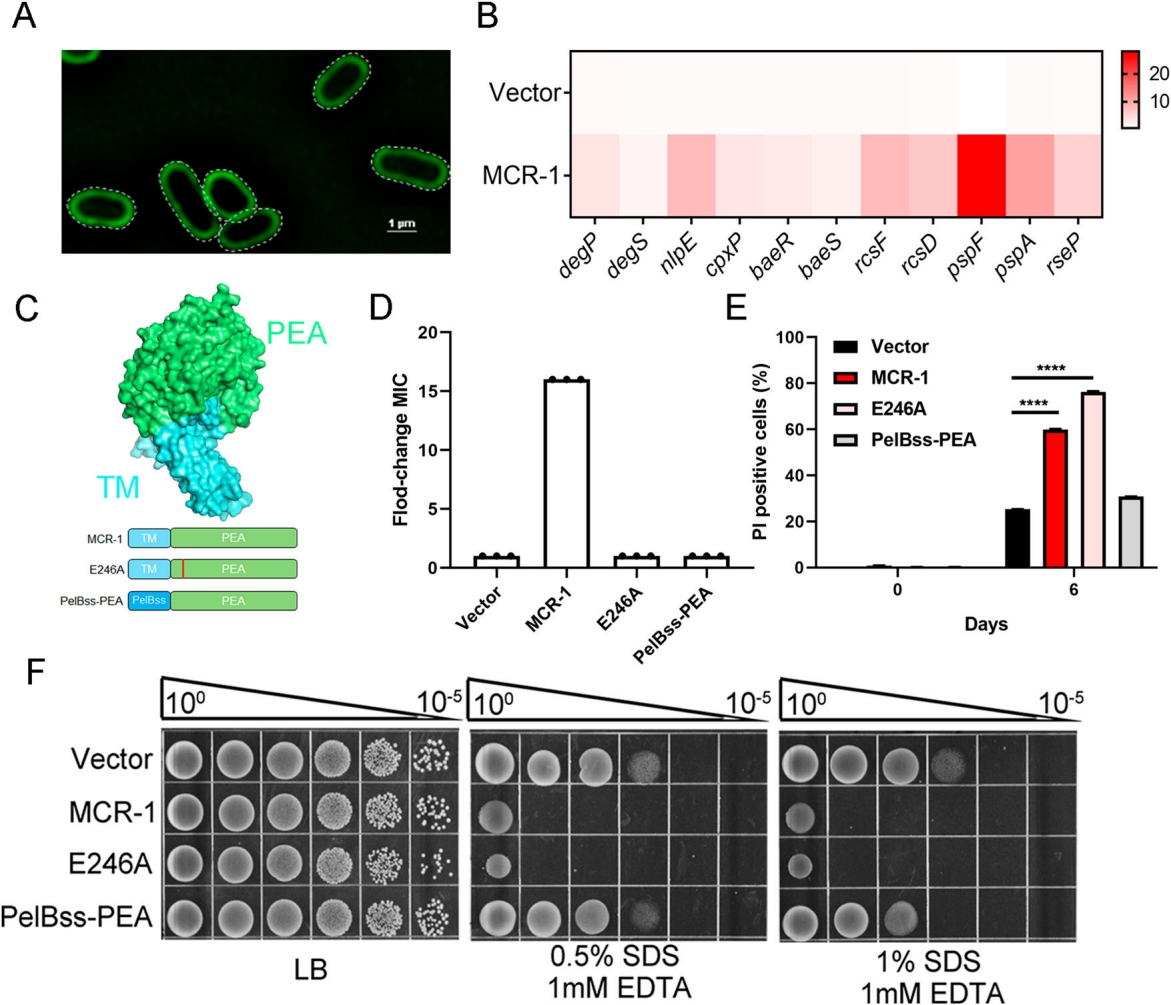
MCR-1 with catalytic domain deletion failed to rescue OM permeability defect and cell death (A) Super-resolution images of BW25113 carrying MCR-1-sfGFP, illustrating that MCR-1 is evenly distributed on the cell membrane. (B) Heat maps showing the upregulated expression of genes associated with stress response were upregulated in MCR-1-expressing cells. (C) Scheme of the two mutants of MCR-1 (E246A and PelBss–PEA). E246A, a mutant of MCR-1 without catalytic activity; PelBss–PEA combines the PelB signal peptide and the catalytic domain of MCR-1 (PEA). (D) Neither of the two mutants (E246A nor PelBss–PEA) conferred resistance to colistin. (E) Percentage of dead cells was evaluated with flow cytometry. After the indicated times in culture, the cells were stained with PI and washed with PBS before flow cytometry (*n* = 3 biological replicates per group, data are means ± SEM, assessed with two-way ANOVA and Tukey’s multiple comparison test; ****p < 0.0001 compared with Vector group). (F) Serial dilutions of the indicated strains were spotted onto LB agar plates or LB agar plates supplemented with SDS/EDTA. The bacteria were then grown at 37°C.

To understand how MCR-1 impairs OM permeability, we investigated the molecular mechanism underlying this phenomenon. To determine the localization of MCR-1, we labelled the protein with super-folded green fluorescent protein (sfGFP) for super-resolution imaging. As shown in Figure 3(A), MCR-1 localized at the cell membrane. The overexpression of membrane proteins may increase the stress on the cell envelope, possibly influencing the permeability of OM [32]. We suspected a link between MCR-1 and the cell envelope stress response. Specifically, we found that the expression of genes associated with the stress response, including those encoding Cpx (e.g. *degP*, *degS*, *nlpE*, and *CpxP*), Rcs (e.g. *rcsF*, *rseP*, and *rcsD*), Bae (e.g. *baeR* and *baeS*), and Psp (e.g. *pspF* and *pspA*), was upregulated (Figure 3(B)), indicating that MCR-1 activates the envelope stress response. Given its membrane localization and the functional role of MCR-1, we decided to focus on two possible mechanisms: (i) the modification of lipid-A by MCR-1 reduces the integrity of the cell envelope; and (ii) the accumulation of MCR-1 in the bacterial membrane damages the OM permeability barrier.

The first mechanism was interrogated by measuring the OM permeability of E246A, an MCR-1 mutant in which the enzymatic activity of the pEtN transferase was abolished (Figure 3(C)). We confirmed that strain E246A was sensitive to colistin (Figure 3(D)), which is consistent with previous findings [16]. However, cells expressing MCR-1 E246A displayed a higher death rate (Figure 3(E)) and were sensitive to SDS–EDTA (Figure 3(F)). Consistent with this, the envelope stress response was activated in these cells (Figure S6), suggesting that the catalytic activity of MCR-1 has a negligible effect on cell viability.

To determine whether the second mechanism was involved in the OM defect, we generated a mutant expressing PelBss–PEA, a construct that combined the PelB signal peptide for periplasmic localization and the soluble catalytic domain of MCR-1 (PEA) (Figure 3(C)). The OM permeability of the PelBss–PEA-expressing strain was unchanged and its cell death rate was similar to that of the Vector strain (Figure 3(E and F)). These results demonstrated that the membrane localization of MCR-1, but not its catalytic activity, caused defective OM permeability.

### Potential lipid-A-binding pocket affects OM permeability

**Figure 4.**
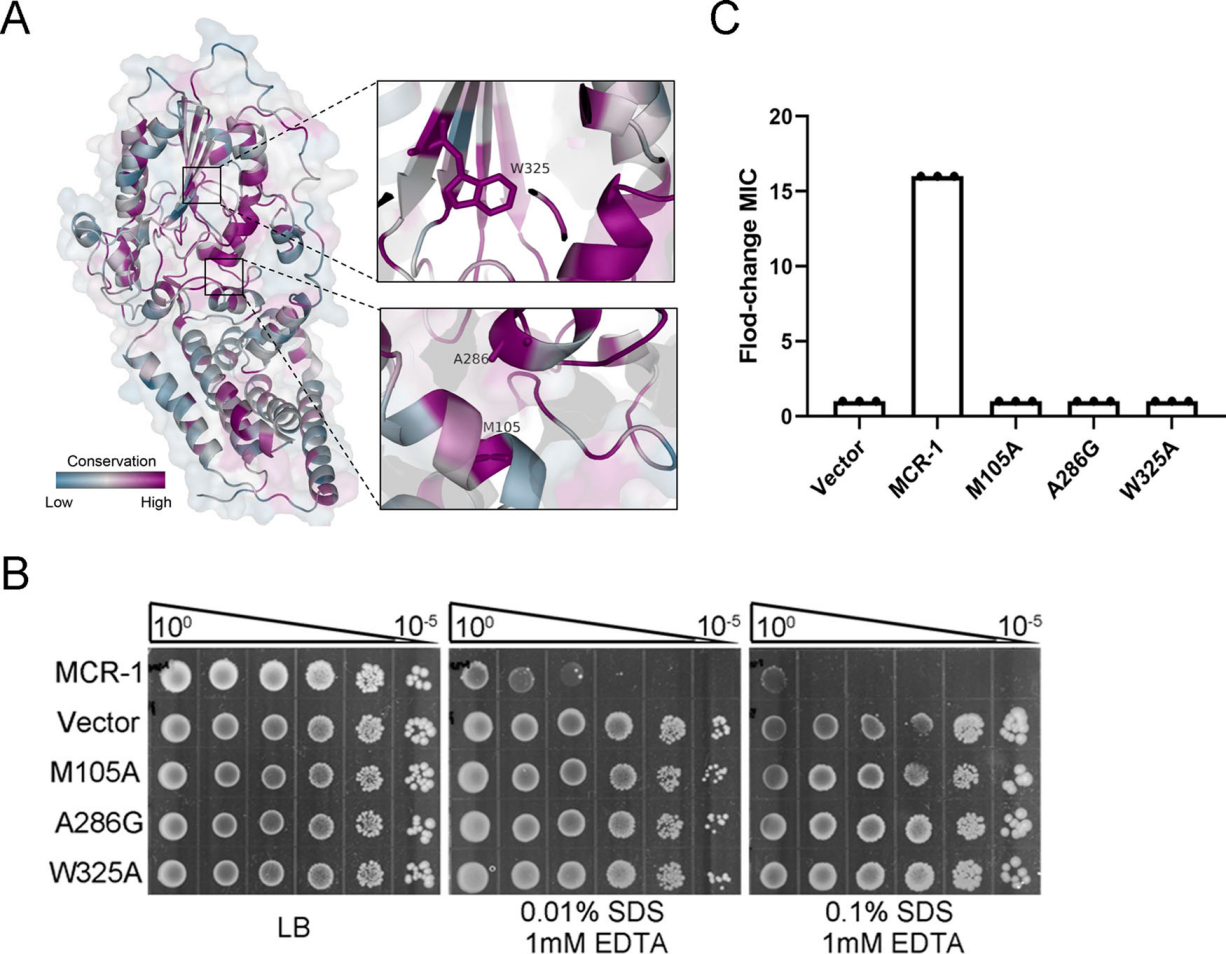
Potential lipid-A-binding pocket in MCR-1 is responsible for increased OM permeability and cell death. (A) Conservation analysis of 103 MCR family proteins; surface and cartoon representations. Three conserved residues in the potential lipid-A-binding pocket are labelled. (B) Functional assay of three mutations in MCR-1 essential for colistin resistance. A representative result from three independent experiments is shown. (C) Serial dilutions of the indicated strains were spotted onto LB agar plates or LB agar plates supplemented with SDS/EDTA. The bacteria were then grown at 37°C.

The membrane localization of MCR-1 involves its transmembrane domain and a linker domain. A recent structural study showed that the full-length structure of PbgA, an enigmatic inner membrane protein that is similar to MCR-1, assembles as a homotetrameric complex, and its lipid-A-binding motif interacts with LPS, affecting OM permeability [33]. Very recently, Xu et al. [34] showed that the potential lipid-A-binding pocket of MCR-3 facilitates phenotypic colistin resistance. Because the crystal structure of full-length MCR-1 is unknown, the structure of MCR-1 was modelled using the EptA (PDB: 5FGN.1.A) enzyme as the structural template to determine whether MCR-1 contains a lipid-A-binding pocket. We next aligned all 103 MCR family protein sequences from the NCBI database to identify the conservation within MCR-1. We found that the catalytic domain is highly conserved (Figure 4(A)). It is noteworthy that the positions of M100, A278, and W317 in MCR-3 corresponded to the M105, A286, and W325 residues in MCR-1, and that these amino acids, located in the lipid-A-binding cavity, were highly conserved in MCR-1 and MCR-3 (Figure 4(A)), indicating that M105, A286, and W325 in MCR-1 were critical for lipid-A-binding.

We introduced point mutations into the potential lipid-A-binding pocket of MCR-1 and evaluated the OM permeability of cells expressing the mutants. Interestingly, the strains expressing MCR-1 mutation M105A, A286G, or W325A displayed OM permeability similar to that of the Vector strain (Figure 4(B)), indicating the lipid-A-binding pocket is responsible for the defect in OM permeability. Moreover, these three mutants were sensitive to colistin (Figure 4(C)) and displayed reduced cell death rates (Figure S7). These data demonstrated that the potential lipid-A-binding pocket of MCR-1 affected the OM permeability of the host cell.

### Lipid remodelling is a major cause of reduced viability

MCR-1 induces both a defect in OM permeability and bacterial shrinkage, which prompted us to hypothesize that the Tol–Pal system is involved in these phenotypes, because the Tol–Pal system is required for cell shrinkage and the maintenance of OM integrity [35,36]. To test this, we first examined the protein expression level of the Tol–Pal system. Compared with the Vector cells, the expression of *tolA*, *pal*, and *tolR* was upregulated in the MCR-1-expressing strain (Figure S8A). However, the deletion of *pal* did not restore OM permeability (Figure S8B), suggesting that the Tol–Pal system was not required for the MCR-1-mediated OM permeability defect.

**Figure 5.**
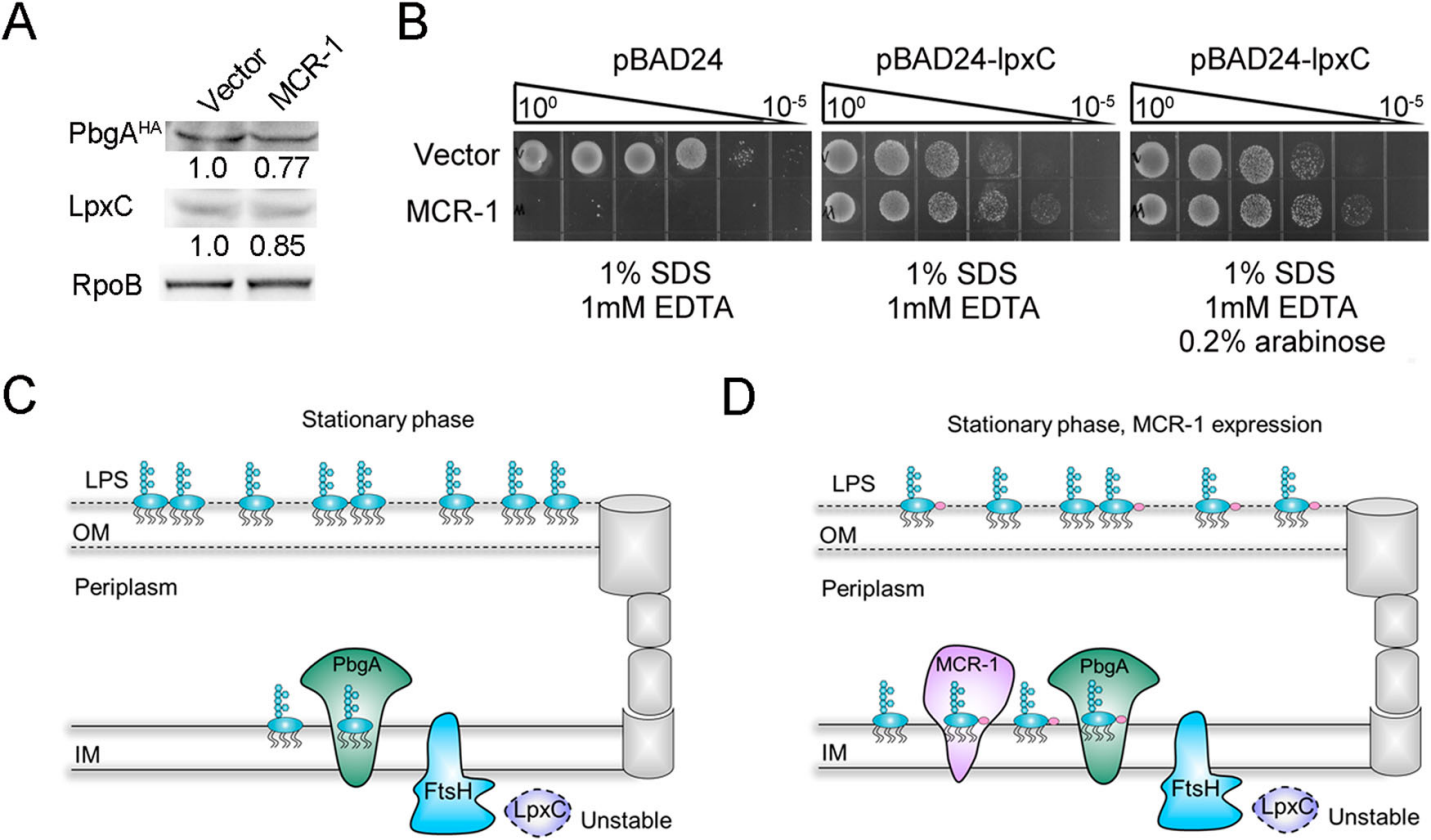
MCR-1-induced lipid remodelling contributes to OM permeability defects. (A) LpxC and PbgA^HA^ proteins in whole-cell lysates of stationary-phase cells were measured with western blotting using anti-LpxC and anti-HA antibodies, respectively. RNA polymerase subunit beta (RpoB) was used as the loading control. (B) Serial dilutions of the indicated strains were spotted onto LB agar plates or LB agar plates supplemented with SDS/EDTA in the presence or absence of arabinose. The bacteria were then grown at 37°C. (C) Model of how MCR-1 controls the outer membrane permeability defect. In the stationary-phase cells, accumulated periplasmic LPS tends to bind to the PbgA–LapB complex, which promotes the degradation of LpxC by FtsH. (D) Illustration of the MCR-1-mediated phenotype during stationary phase.

The disruption of lipid homeostasis affects OM permeability and cell death [30]. Therefore, we investigated whether MCR-1 damages the OM permeability barrier by disrupting lipid homeostasis. To test our hypothesis, we generated a strain in which PbgA, a sensor of periplasmic lipid-A [37], was labelled with hemagglutinin (HA). Intriguingly, the level of PbgA protein was lower in the MCR-1-expressing cells than in the Vector cells (Figure 5(A)), indicating that MCR-1 disrupted lipid homeostasis. The expression of LpxC, which catalyzes the first committed step in lipid-A synthesis [37], was also determined and was lower in MCR-1-positive cells than in the Vector cells (Figure 5(A)). This result strongly suggested that MCR-1 affected lipid homeostasis.

To confirm this observation, an LpxC-overexpressing strain was generated to test its OM permeability in the presence or absence of MCR-1. The OM permeability defect caused by MCR-1 was abolished when LpxC was overexpressed, even though a low level of LpxC complement without the induction of arabinose (Figure 5(B)). This could be explained by the leaky regulation of LpxC expression by the arabinose promoter. In fact, in stationary-phase cells, the LPS accumulated in the periplasm tended to bind the PbgA–LapB complex, which promoted the degradation of LpxC by FtsH [33] (Figure 5(C)). We inferred that in the presence of MCR-1, the LPS–PbgA–LapB complex increased in the cell periplasm, and accelerated the FtsH-mediated regulation of LpxC levels (Figure 5(D)). This notion suggested that MCR-1-dependent lipid remodelling increased OM permeability, ultimately resulting in cell death during the stationary phase. This was a plausible explanation for the reduced prevalence of *mcr-1* in humans, animals, and the environment after the withdrawal of colistin from use.

It has been reported that LpxC mutants, such as V37G [38] and lpxC_*306fs_ [39], increase cellular LpxC levels, which may restore OM permeability defect impaired by MCR-1. These mutations, which have been detected in *mcr-1*-positive isolates, should be of great concern. We determined the amino acid sequences of LpxC amplified from 688 *mcr-1*-positive isolates and only nine isolates contained an LpxC mutant. Eight variants were identified: A13T, A305 deletion (A305^−^), D167N, R237H, S211T, S301L, V138I, and V93I (Figure S9), but none of these mutants was reported to increase cellular LpxC levels.

## Discussion

In this study, we sought to better understand the viability of *mcr-1*-positive Gram-negative bacteria in the stationary phase. Our data suggested that MCR-1 damaged the OM permeability barrier in *E. coli* and *K. pneumonia,* the major host species of *mcr-1*. The deletion of *mcr-1* from dominant clinical plasmids IncX4 and IncHI2 also restored the OM permeability defect. Consequently, *mcr-1*-positive bacteria exhibited shrinkage and cell death in nutrient-limited stationary phase. Importantly, the ability of *mcr-1*-positive *E. coli* to recover from starvation under more favourable growth conditions was reduced. These findings may explain why the prevalence of *mcr-1* was reduced in previous studies. We also found that the potential lipid-A-binding pocket of MCR-1, but not its catalytic activity, is responsible for the OM permeability defect in *mcr-1*-positive cells. Furthermore, lipid homeostasis was disrupted by MCR-1, and the overexpression of LpxC, which catalyses the first step in lipid-A synthesis, completely reversed the MCR-1-mediated OM permeability defect. Overall, our findings indicated that the lipid remodelling caused by MCR-1 impaired the OM permeability barrier, resulting in cell shrinkage and death during stationary phase.

A recent study reported that *E. coli* BW25113 carrying an IncI2-type plasmid, pHNSHP45, imposed a fitness cost compared with BW25113 carrying pHNSHP45 Δ*mcr-1*. The deletion of *pcnR*, which increased the plasmid copy number, generated a high cost on the host. Remarkably, the reduced fitness of the *pcnR* mutant was abrogated by disrupting the *mcr-1* gene [40]. These results suggest that MCR-1 exerts an effect on bacterial fitness. Consistent with this, the overexpression of *mcr-1* has been shown to reduce bacterial fitness, retard the growth rate, and disrupt the structural integrity of the membrane. In this study, we used a low-copy number plasmid and the native promoter to control *mcr-1* expression and found that MCR-1 damaged the OM permeability barrier. This phenotype was also observed in BW25113 carrying IncX4 or IncHI2, and even in *mcr-1*-positive *K. pneumonia*. It was therefore likely that MCR-1-mediated OM permeability defect is ubiquitous across host bacteria. Interestingly, as cells entered into stationary phase, *E. coli* carrying IncX4-*mcr-1* [41] or IncI2-*mcr-1* [40] imposes a fitness cost compared to *mcr-1*-negative *E. coli*, and this fitness cost significantly increased in a time-dependent manner. Our data further indicated that the viability of *mcr-1*-positive bacteria may be compromised in the stationary phase. In the present study, short-term bacterial survival was studied in the stationary phase (6 days). It should be noted that many microorganisms face far longer periods of starvation in their natural environments [42]. Therefore, it could be explained postulated that the prevalence of *mcr-1* was reduced in humans, animals, and the environment due to this fitness cost.

What is the mechanism behind MCR-1-induced cell death during stationary phase? It has been proposed that bacterial shrinkage results from the loss of cellular material into the extracellular milieu [43]. Shi *et al.* [28] previously demonstrated that cell shrinkage is primarily driven by dehydration. Notably, it has been shown that the expression of *mcr-1* reduces bacterial tolerance to high salt stress [44], indicating a link between MCR-1 and membrane permeability. It is well known that protein aggregation has mostly been seen as a symptom of stress and damage [45], while a recent study infers that protein aggregation as a broad protective effect, conferred robustness to other proteotoxic stresses, such as streptomycin exposure or oxidative stress [46]. Enhanced protein aggregation was also observed in the MCR-1-expressing cells, which suggested that cellular damage accumulates in the stationary phase. Indeed, protein aggregation could be dissolved by the protein quality control network once bacteria recovered from stressful conditions [47]. However, the ability of cells to recover from starvation after nutrient supplementation was reduced. Hence, it was likely that cell death was due to the irreversible damage caused by MCR-1 during stationary phase.

**Figure 6.**
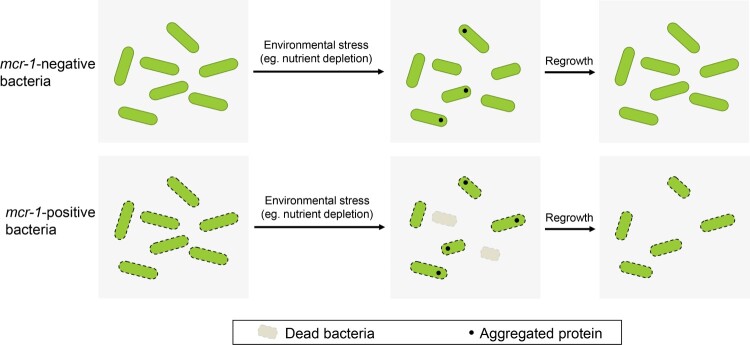
Model summarizing the mechanism underlying the survival and regrowth of *mcr-1*-positive bacteria in the stationary phase. We propose that *mcr-1*-positive bacteria, with increased OM permeability, exhibited reduced viability during stationary phase. Unlike *mcr-1*-negative bacteria, the ability of the cells to recover from stationary phase under improved conditions was also reduced, which may arise from the irreversible damage caused by MCR-1.

Lipid A is synthesized on the inner membrane of Gram-negative bacteria [48], and this molecule is subsequently flipped into the periplasm and either transported to the OM [49]. To adapt to environmental stress, Gram-negative bacteria regulate LPS content on their OM [50]. For example, under stressful conditions, inner membrane protein PbgA is able to detect periplasmic LPS molecules, then interacts with LapB, an adaptor for the FtsH protease. This event may prompt FtsH to degrade LpxC, the rate-limiting LPS biosynthesis enzyme [51]. Our findings suggested that the LPS accumulated in the periplasm of MCR-1-expressing cells increases OM permeability, resulting in cell death, during stationary phase. Several lines of evidence supported this conclusion. First, to clarify the mechanism of the MCR-1-mediated OM permeability defect, we initially hypothesized that pEtN modification of lipid-A damaged OM, because the overexpression of MCR-1 caused substantial changes to the morphology and physicochemical properties of the OM [52]. However, the OM permeability was not restored in *E. coli* E246A, an MCR-1 mutant without pEtN transferase activity. Furthermore, the deletion of the *pal* gene was unable to restore the phenotype caused by MCR-1. It stands to reason that a yet unidentified mechanism is involved in MCR-1-regulated OM permeability. Second, we found that MCR-1 mutations expected to disrupt its binding to lipid-A in the periplasm restored OM permeability, suggesting that MCR-1 expression disturbs lipid homeostasis. The disruption of lipid homeostasis is known to increase OM permeability [30,37]. For instance, Randi et al. demonstrated that the depletion of phospholipids in the OM was the major cause of the OM defect [37]. The distribution of lipid-A along the periplasmic leaflet also affected the OM barrier. Third, during stationary phase, PbgA and LpxC levels were reduced in the MCR-1-expressing cells, and the overexpression of LpxC completely rescued the MCR-1-mediated OM permeability defect. According to a previous model, during stationary phase, increased LPS–PbgA–LapB complex promotes the degradation of LpxC by FtsH [33]. It was possible that the membrane protein MCR-1 bound to lipid-A, leading to accumulation of LPS within the inner membrane. This event may increase the LPS–PbgA–LapB complex along the periplasmic leaflet, ultimately promoting the degradation of LpxC by FtsH (Figure 6).

There are a few caveats to our study that warrant discussion. First, we only investigated the fate of MCR-1-expressing cells in the stationary phase, and as a result, we did not explore the impact of changes in pH or temperature, which may occur in real life. Furthermore, other microbial cohabitants and host factors should be taken into consideration. Exploring these interactions will be an interesting direction of future research. Second, although our data showed that MCR-1 had a potential lipid-A-binding pocket, the binding motif is unknown. Recently, Thomas *et al*. identified a lipid-A-binding motif in the PbgA protein and found that an optimized peptide derived from this motif inhibited the growth of diverse Gram-negative bacteria [33]. Investigating the lipid-A-binding motif in MCR-1 will also be an interesting direction of future research. Third, although our model rationalized the mechanism underlying the reduced prevalence of *mcr-1* and indicated that MCR-1 disrupted lipid hemostasis, key questions persist. How the MCR-1–lipid-A complex alters the PbgA–LapB interaction and modulates LpxC stability remains unknown. Therefore, the detailed mechanism by which MCR-1 affected lipid-A transport requires further investigation. the impact of MCR-1 on bacterial physiology.

In conclusion, our study revealed that MCR-1 plays an important role in bacterial physiology where, MCR-1 disrupts lipid homeostasis leading to OM permeability defects. Overall, our data provided new insights into the mechanisms underlying the reduced prevalence of *mcr-1* and suggested potential strategy for eliminating drug-resistant bacteria.

## Supplementary Material

Supplemental Material

Supplemental Material

Supplemental Material

Supplemental Material

Supplemental Material

Supplemental Material

Supplemental Material

Supplemental Material

Supplemental Material

Supplemental Material

Supplemental Material

Supplemental Material

Supplemental Material
