# Supplementary figures and images for: MCR-1-dependent lipid remodelling compromises the viability of Gram-negative bacteria
Source: Emerg Microbes Infect. 2022 Apr 28;11(1):1236–49. doi: 10.1080/22221751.2022.2065934 (PMC9067951; doi:10.1080/22221751.2022.2065934)

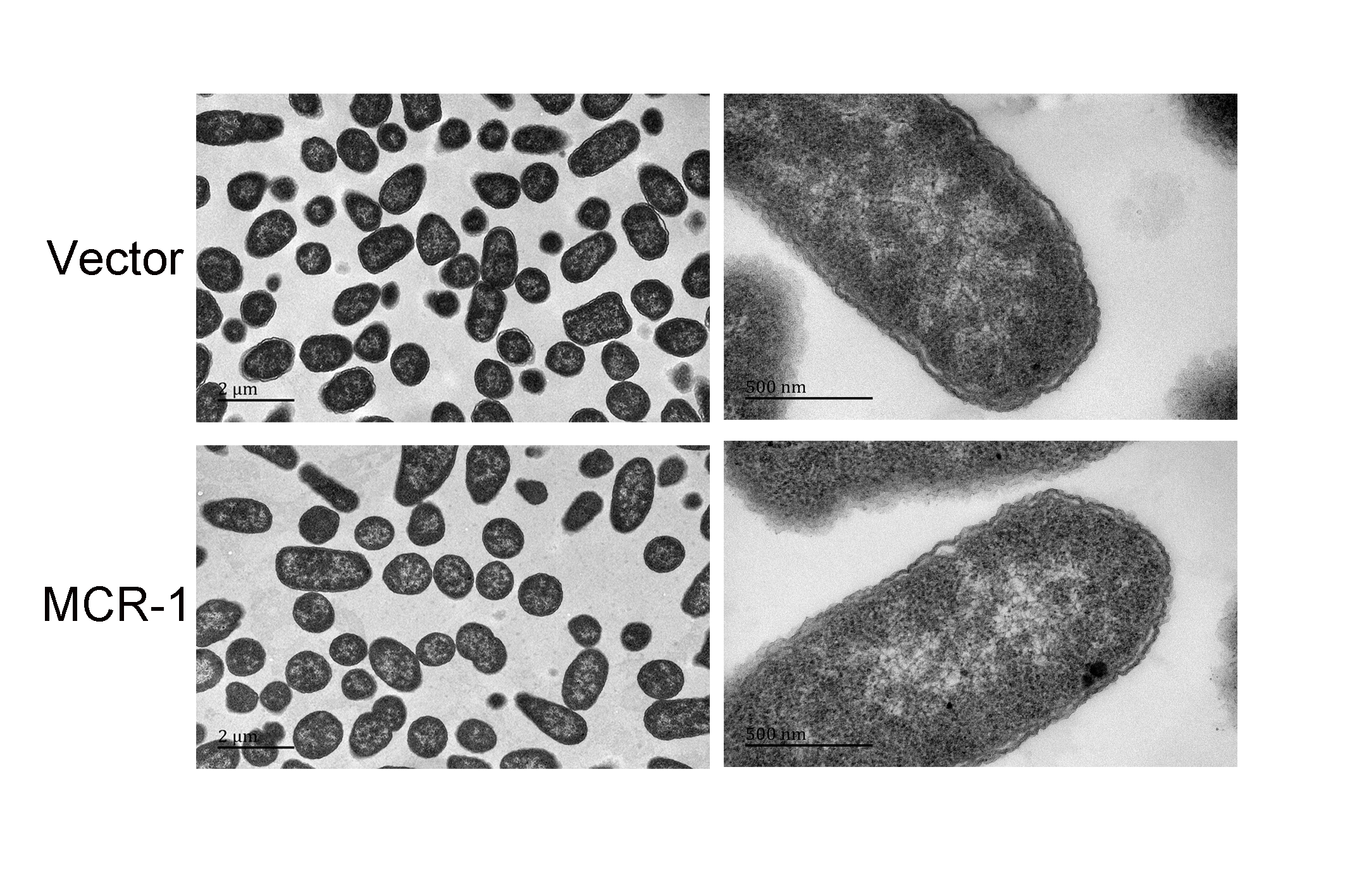

Supplement: Supplemental Material [file TEMI_A_2065934_SM5477.tif]

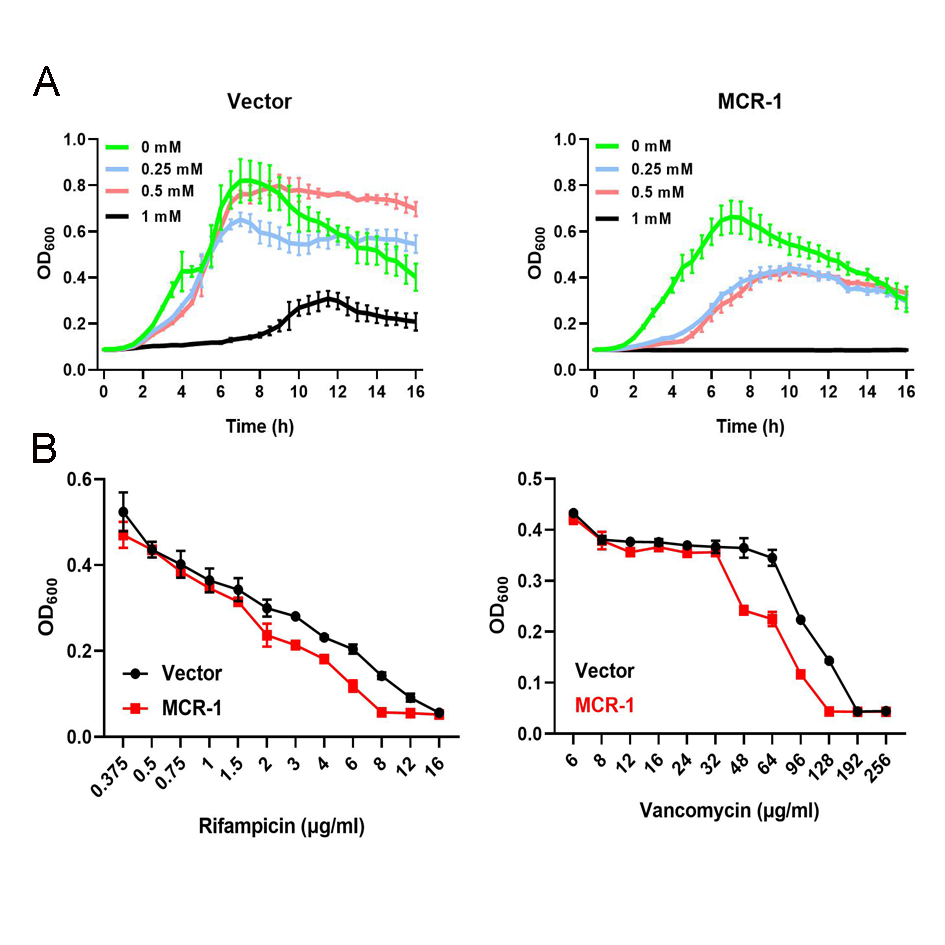

Supplement: Supplemental Material [file TEMI_A_2065934_SM5478.tif]

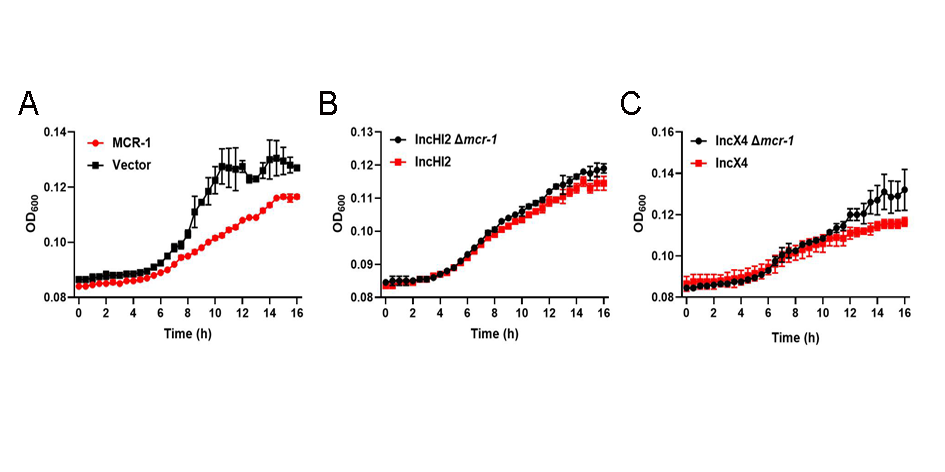

Supplement: Supplemental Material [file TEMI_A_2065934_SM5479.tif]

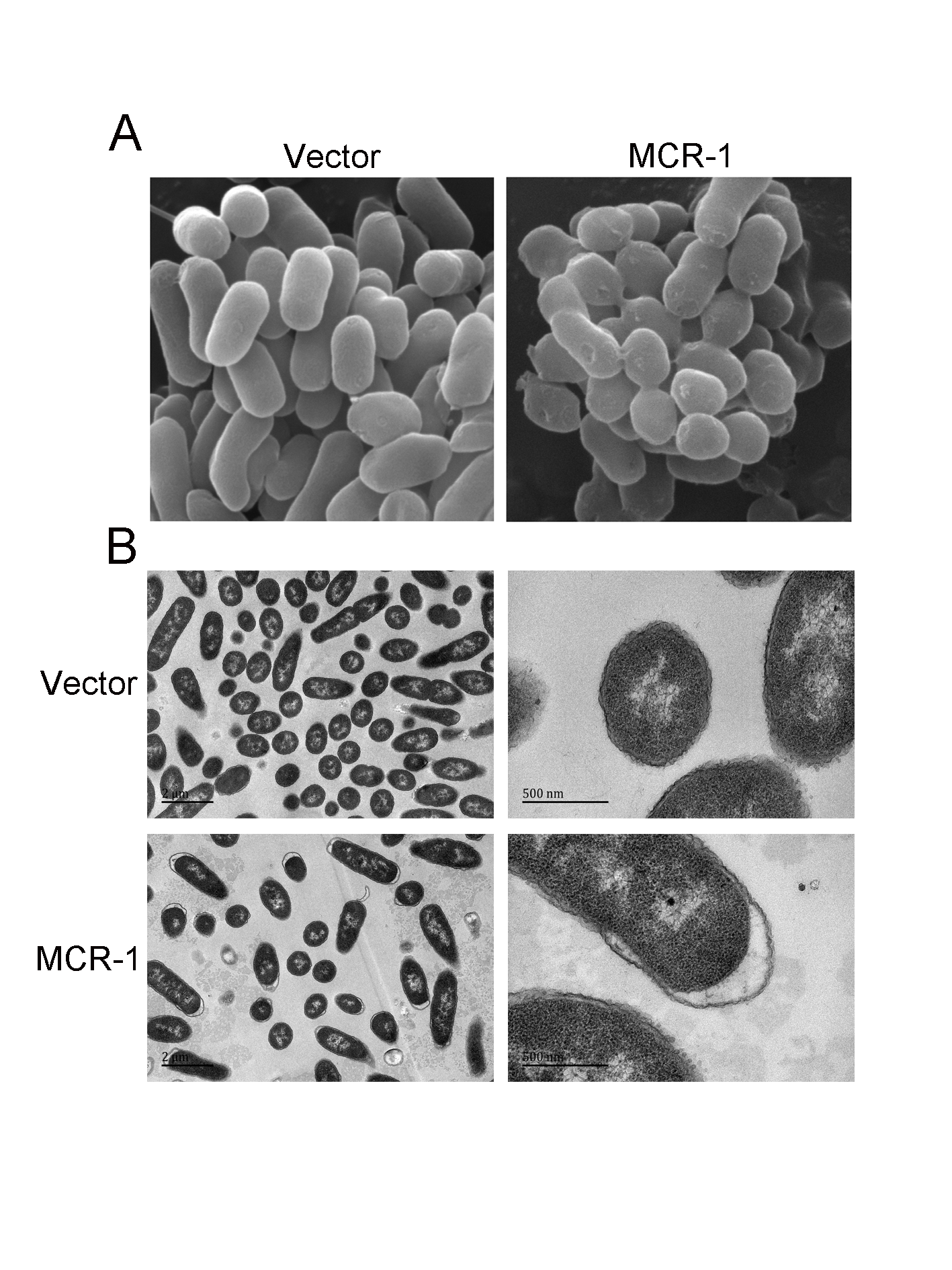

Supplement: Supplemental Material [file TEMI_A_2065934_SM5480.tif]

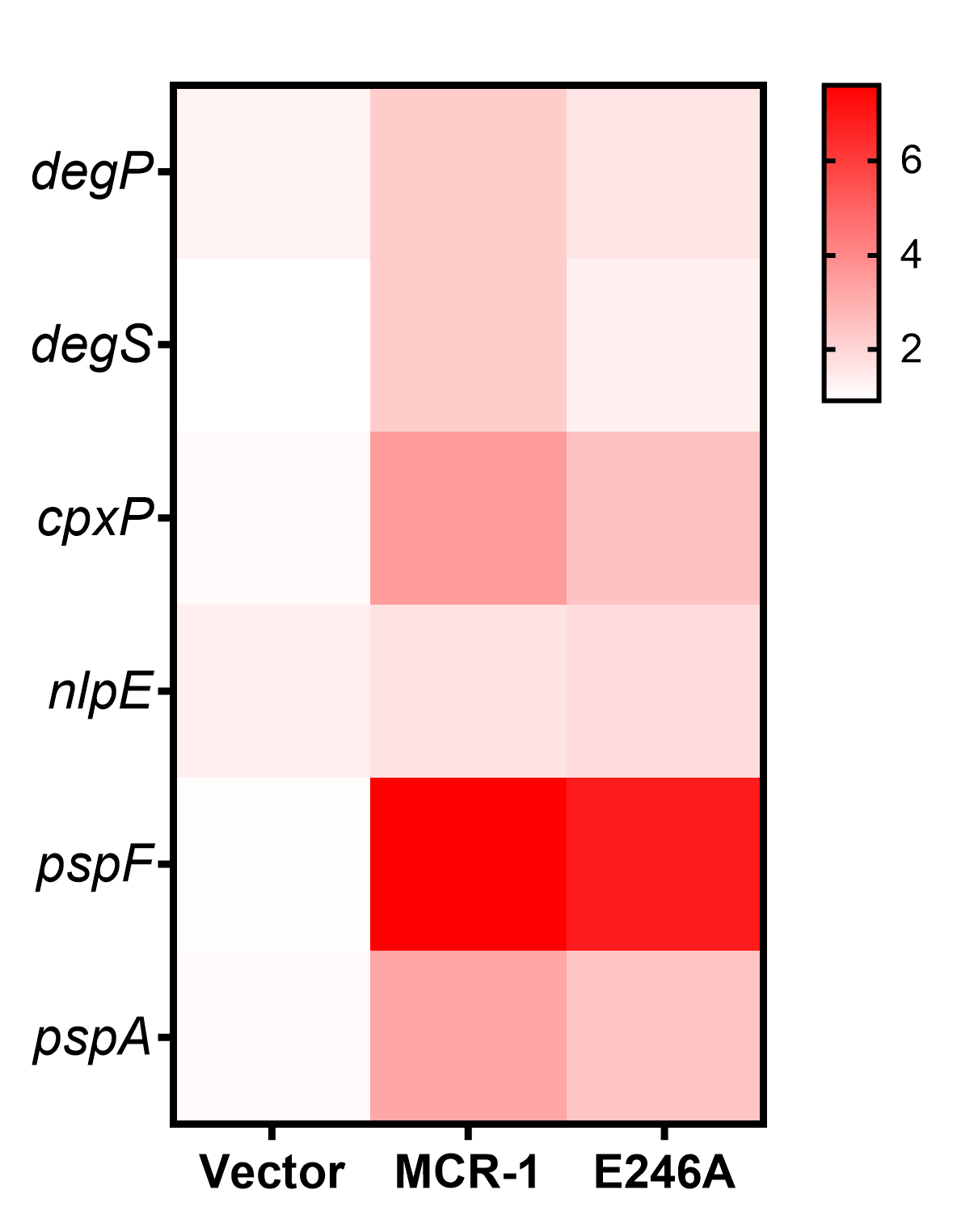

Supplement: Supplemental Material [file TEMI_A_2065934_SM5484.tif]

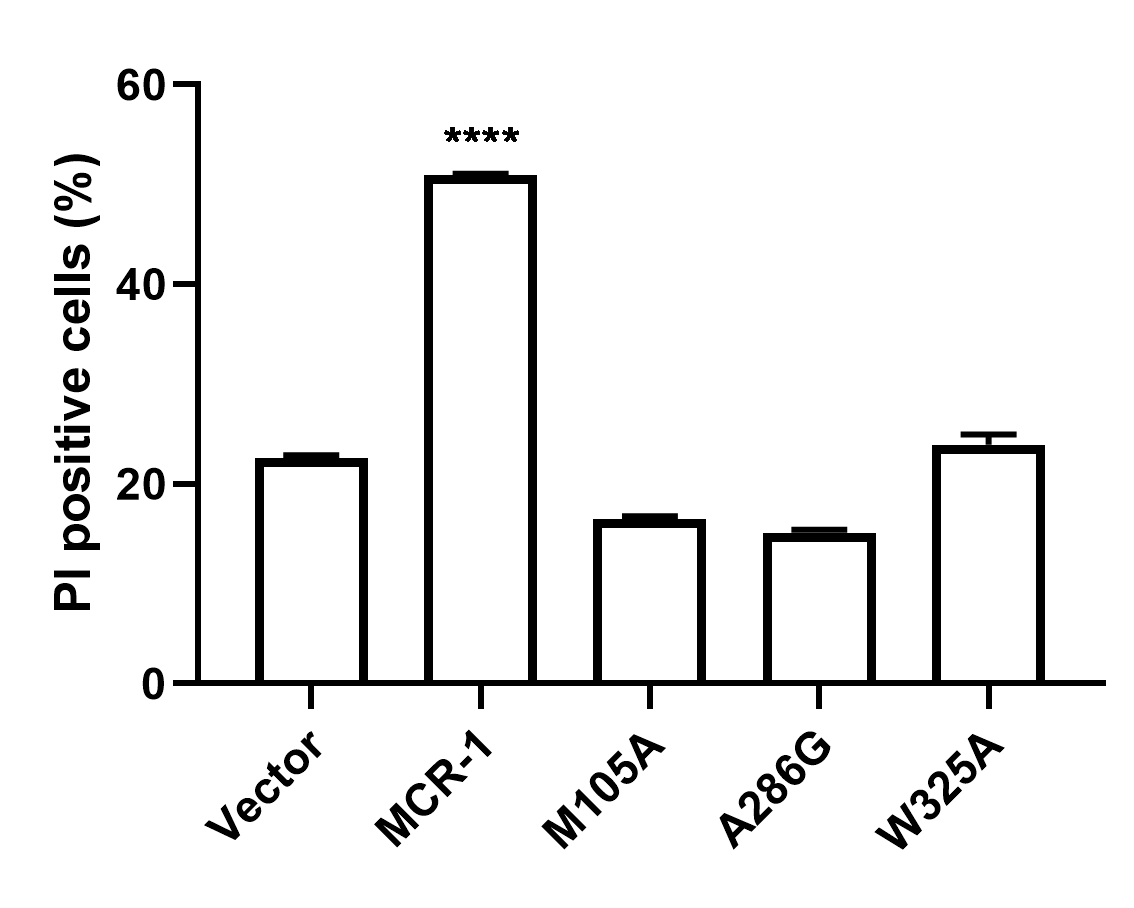

Supplement: Supplemental Material [file TEMI_A_2065934_SM5487.tif]

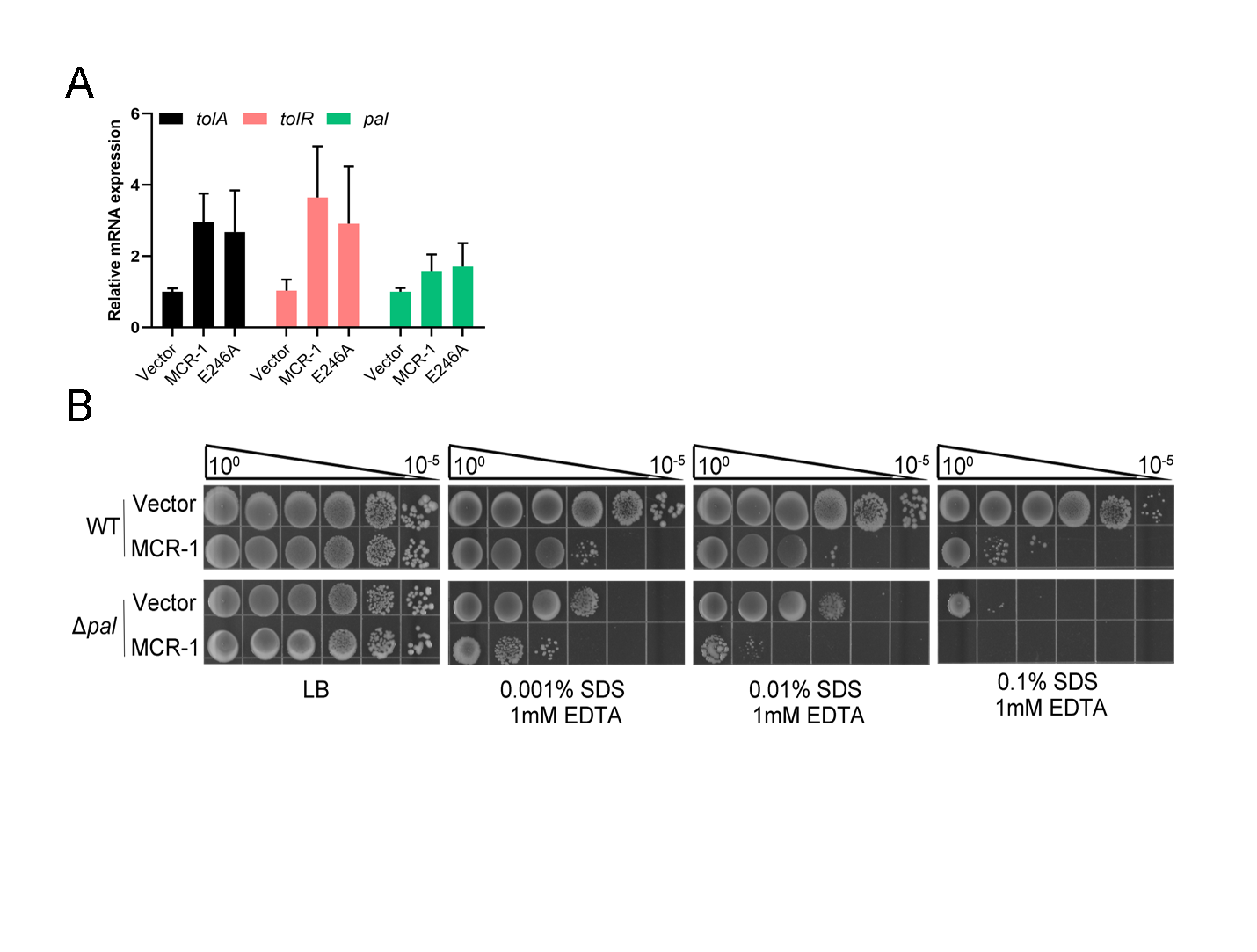

Supplement: Supplemental Material [file TEMI_A_2065934_SM5488.tif]

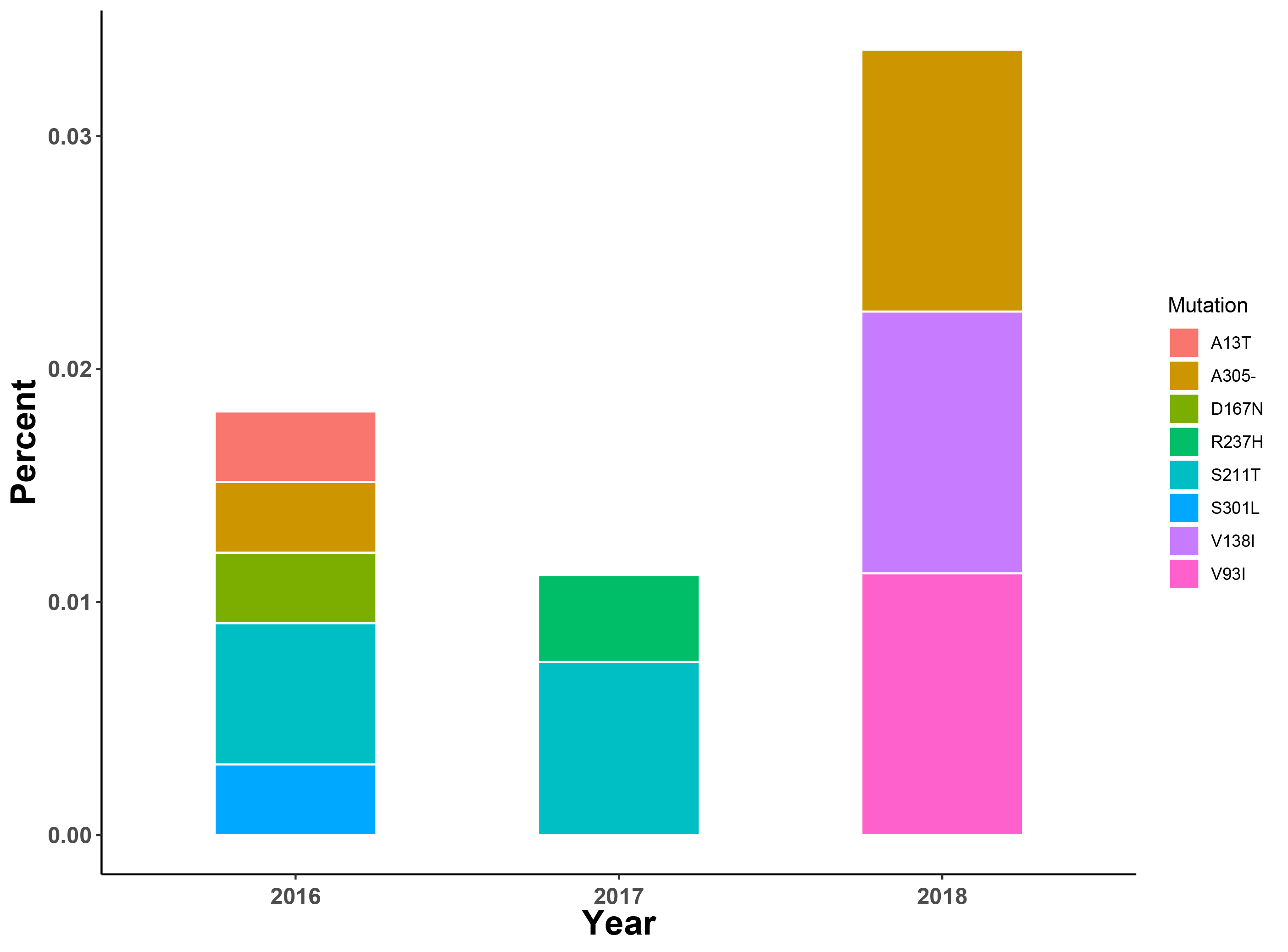

Supplement: Supplemental Material [file TEMI_A_2065934_SM5597.tif]

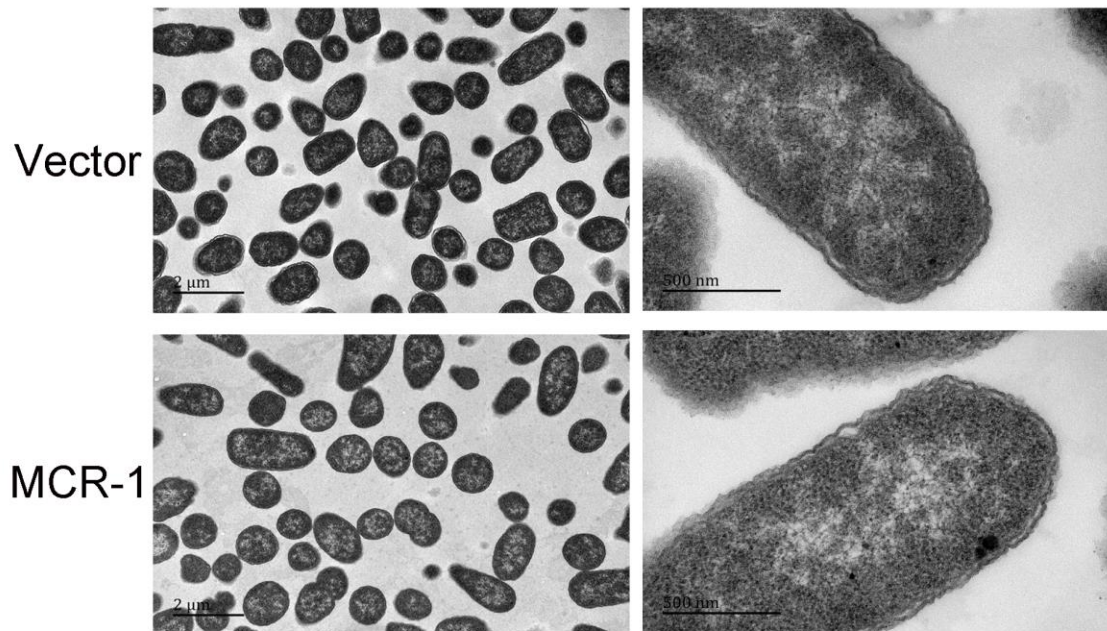

Figure s1

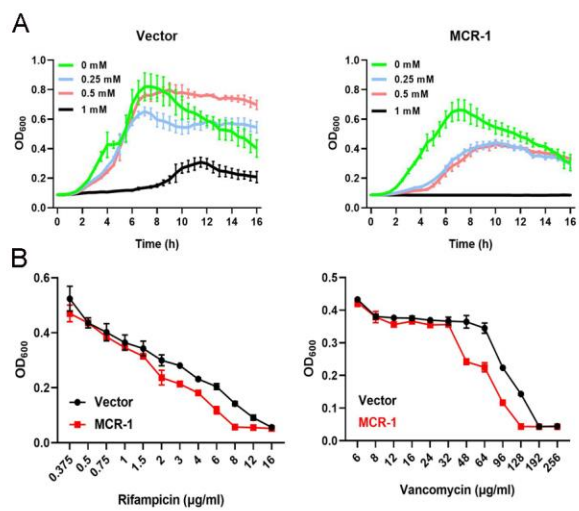

Figure s2

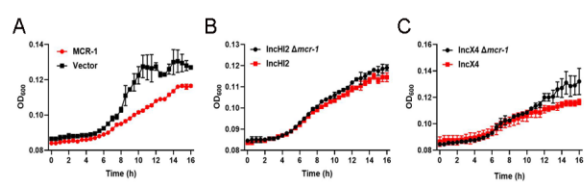

Figure s3

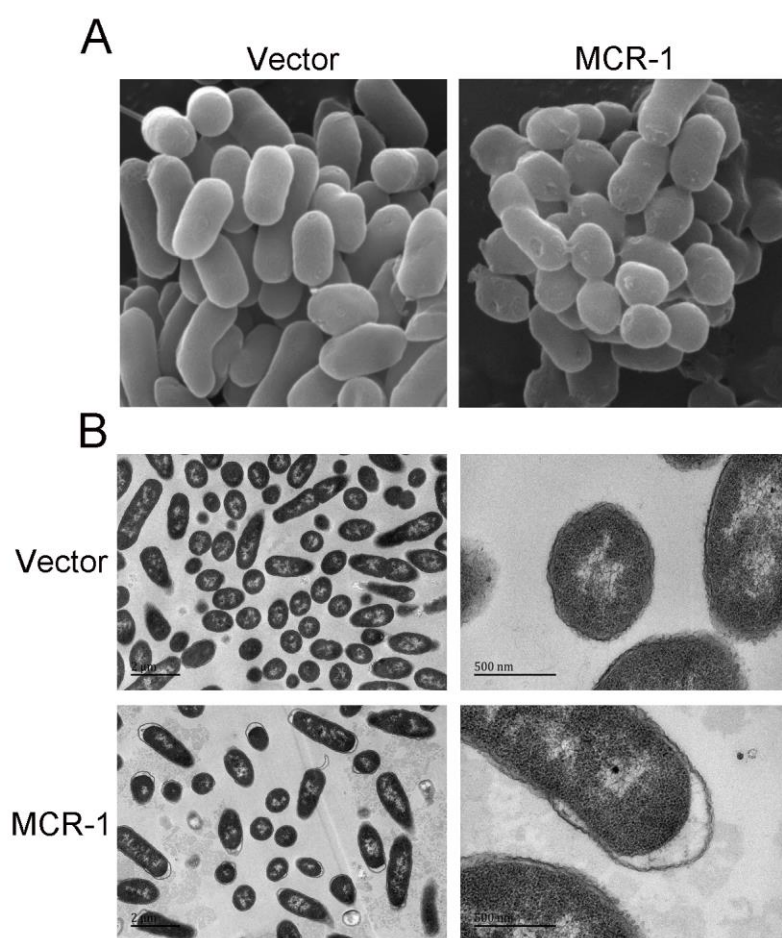

Figure s4

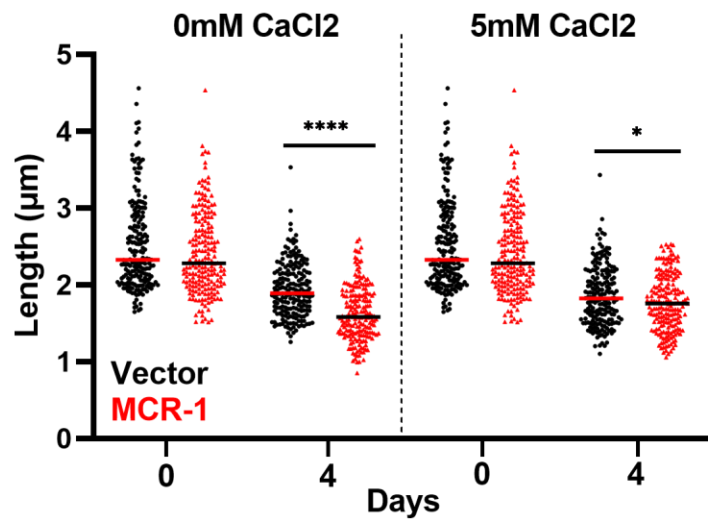

Figure s5

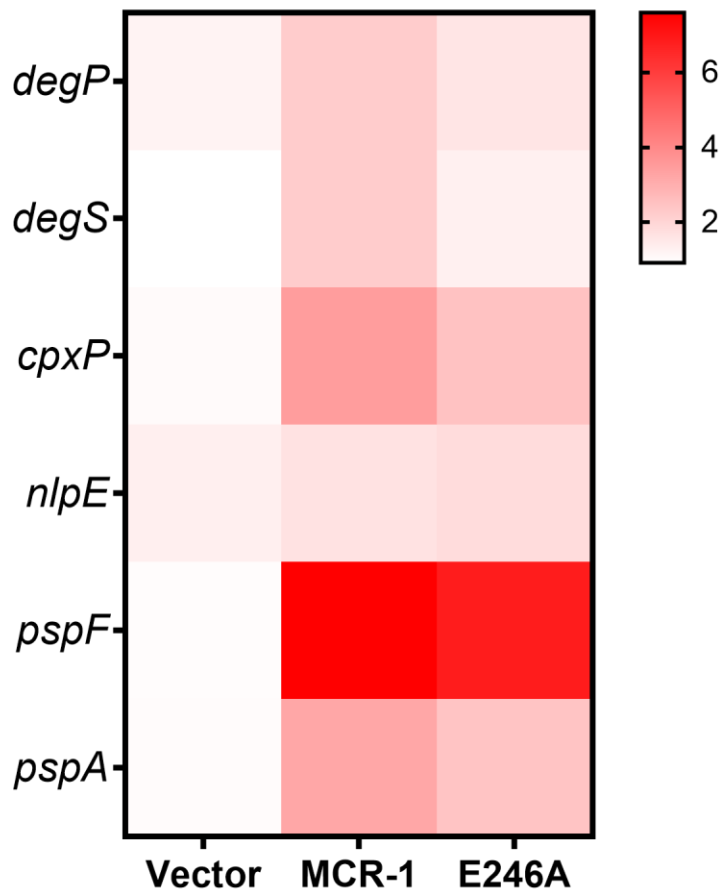

Figure s6

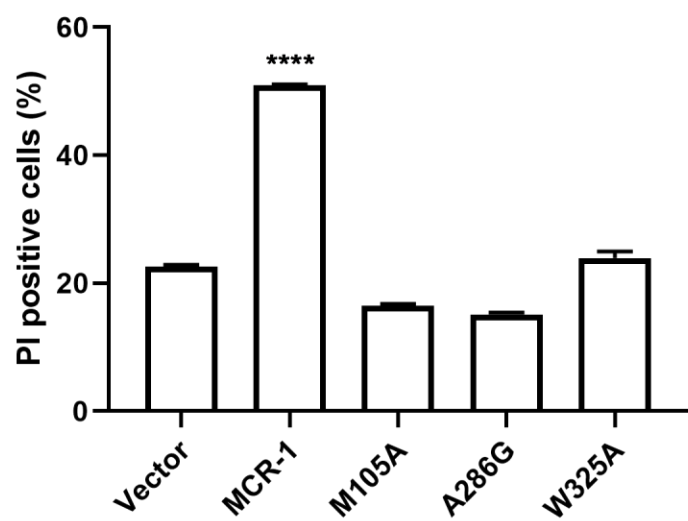

Figure s7

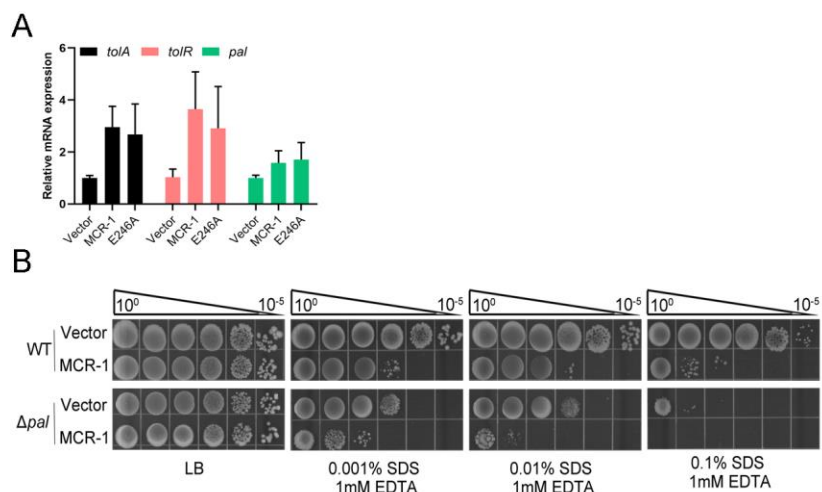

Figure s8

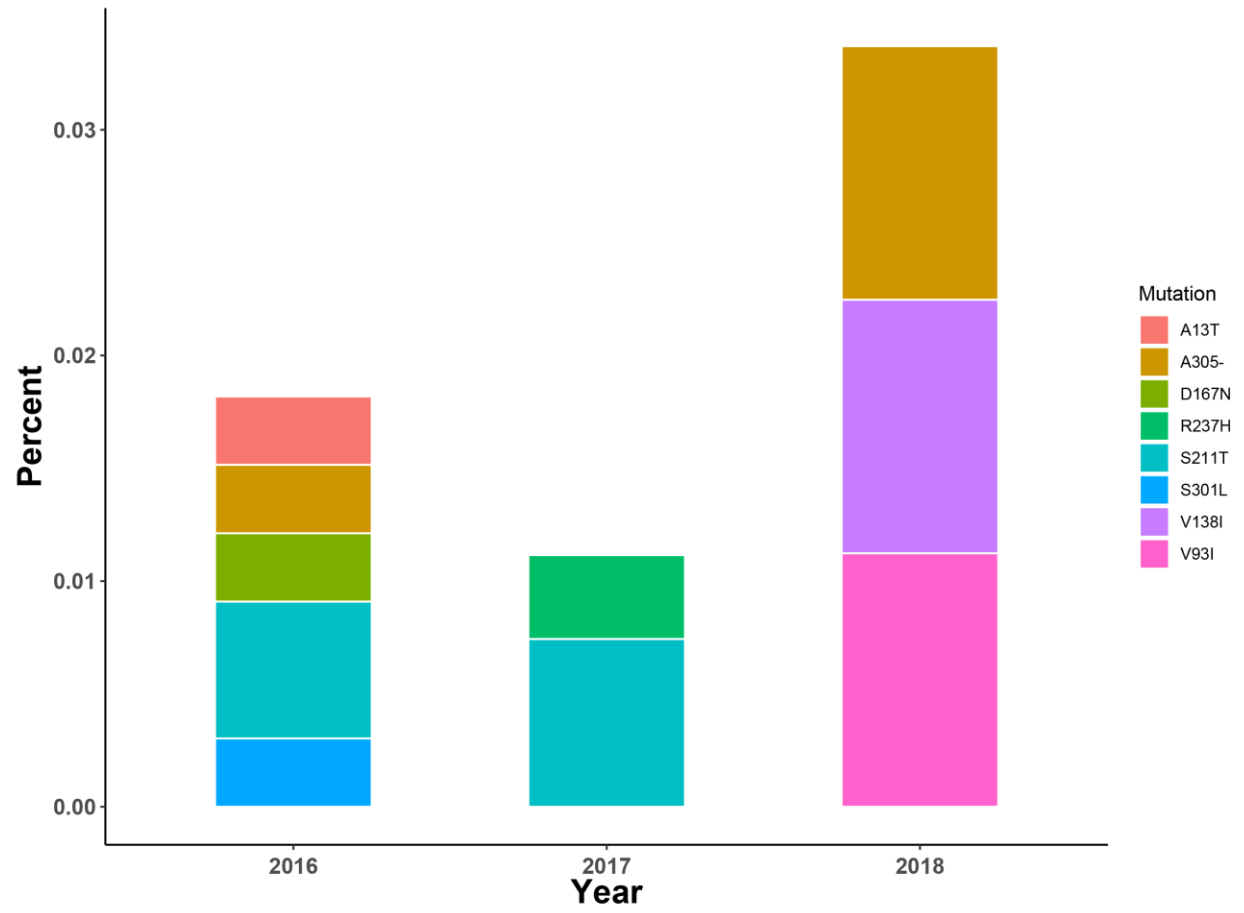

Figure s9

Supplement: Supplemental Material [file TEMI_A_2065934_SM7695.pdf]
