# Supplementary material for: MCR-1-dependent lipid remodelling compromises the viability of Gram-negative bacteria
Source: Emerg Microbes Infect. 2022 Apr 28;11(1):1236–49. doi: 10.1080/22221751.2022.2065934 (PMC9067951; doi:10.1080/22221751.2022.2065934)
Supplement: Supplemental Material [file TEMI_A_2065934_SM5481.doc]

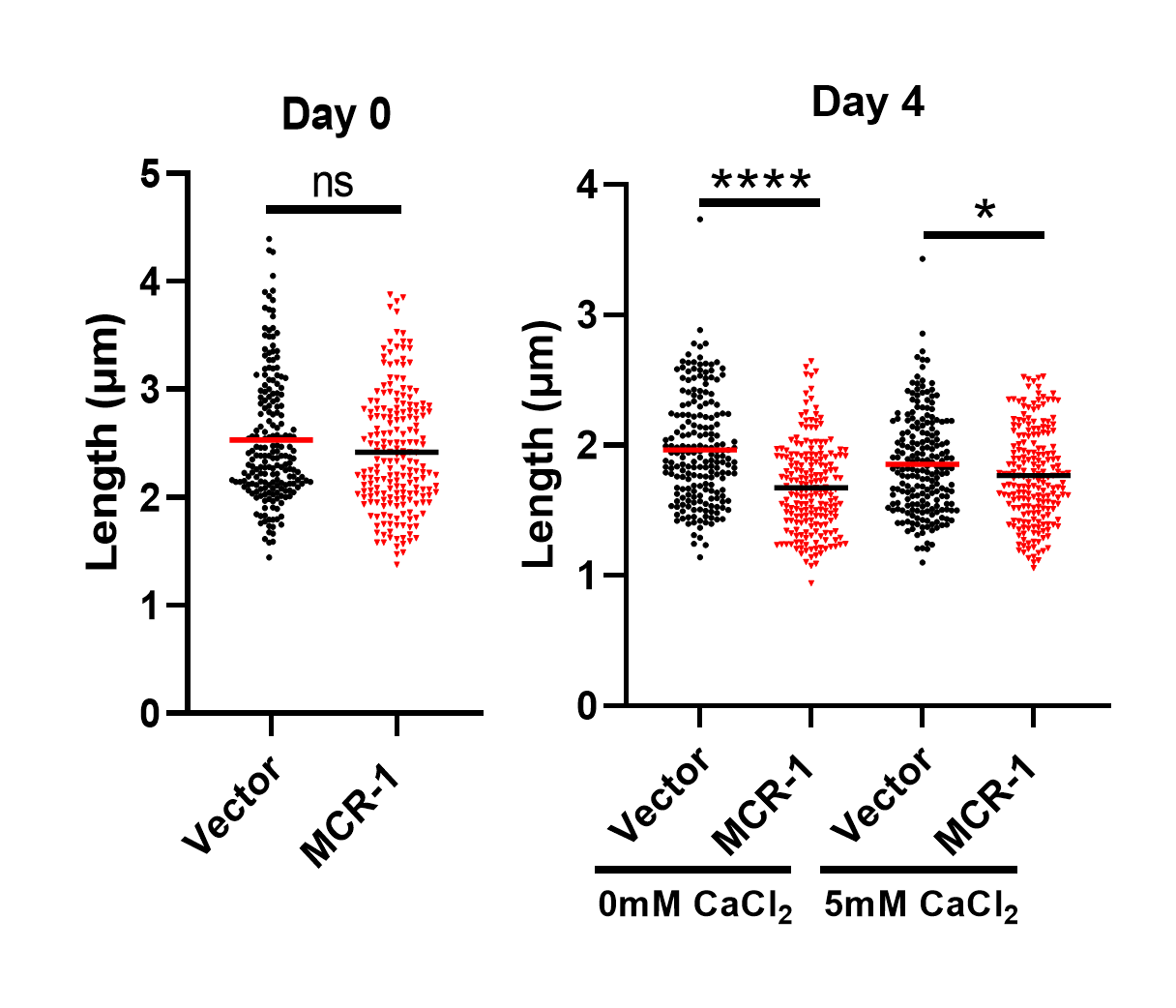


**Figure S5. Ca^2+^ partly rescued MCR-1-induced bacterial shrinkage.** Scatterplots of cell length in Vector cells (black) or MCR-1-expressing cells (red) on day 0 and day 4. Middle lines represent median values (data are means ± SEM, assessed with an unpaired *t* test, with two-tailed *p* values; **p* < 0.05, *****p* < 0.0001).
