# Supplementary material for: MCR-1-dependent lipid remodelling compromises the viability of Gram-negative bacteria
Source: Emerg Microbes Infect. 2022 Apr 28;11(1):1236–49. doi: 10.1080/22221751.2022.2065934 (PMC9067951; doi:10.1080/22221751.2022.2065934)
Supplement: Supplemental Material [file TEMI_A_2065934_SM7535.docx]

**Table S3 MIC value of antibiotics for *mcr-1*-positive BW25113 and *mcr-1*-negative BW25113**

| Antibiotic | MIC (μg/ml) | | | | |
| --- | --- | --- | --- | --- | --- |
|  | BW25113 | BW25113 carrying IncX4 | BW25113 carrying IncX4 Δ*mcr-1* | BW25113 carrying IncHI2 | BW25113 carrying IncHI2 Δ*mcr-1* |
| Colistin | 1 | 16 | 1 | 16 | 1 |
